# Inter- and intraspecific variation in leaf economic traits in wheat and maize

**DOI:** 10.1093/aobpla/ply006

**Published:** 2018-01-24

**Authors:** Adam R Martin, Christine E Hale, Bruno E L Cerabolini, Johannes H C Cornelissen, Joseph Craine, William A Gough, Jens Kattge, Cairan K F Tirona

**Affiliations:** 1 Department of Physical and Environmental Sciences, University of Toronto Scarborough, Toronto, Canada; 2 Centre for Critical Development Studies, University of Toronto Scarborough, Toronto, Canada; 3 Department of Theoretical and Applied Sciences, University of Insubria, Varese, Italy; 4 Systems Ecology, Department of Ecological Science, Vrije Universiteit, De Boelelaan, The Netherlands; 5 Jonah Ventures, Manhattan, 1908 Bluehills Road, KS, USA; 6 Max-Planck-Institute for Biogeochemistry, Hans-Knöll Straβe, Jena, Germany; 7 German Centre for Integrative Biodiversity Research (iDiv) Halle-Jena-Leipzig, Deutscher Platz 5e, Leipzig, Germany

**Keywords:** Agroecology, functional trait, leaf economics, leaf trait, *Triticum aestivum*, *Zea mays*

## Abstract

Leaf Economics Spectrum (LES) trait variation underpins multiple agroecological processes and many prominent crop yield models. While there are numerous independent studies assessing trait variation in crops, to date there have been no comprehensive assessments of intraspecific trait variation (ITV) in LES traits for wheat and maize: the world’s most widespread crops. Using trait databases and peer-reviewed literature, we compiled over 700 records of specific leaf area (SLA), maximum photosynthetic rates (*A*_max_) and leaf nitrogen (N) concentrations, for wheat and maize. We evaluated intraspecific LES trait variation, and intraspecific trait–environment relationships. While wheat and maize occupy the upper 90th percentile of LES trait values observed across a global species pool, ITV ranged widely across the LES in wheat and maize. Fertilization treatments had strong impacts on leaf N, while plant developmental stage (here standardized as the number of days since planting) had strong impacts on *A*_max_; days since planting, N fertilization and irrigation all influenced SLA. When controlling for these factors, intraspecific responses to temperature and precipitation explained 39.4 and 43.7 % of the variation in *A*_max_ and SLA, respectively, but only 5.4 % of the variation in leaf N. Despite a long history of domestication in these species, ITV in wheat and maize among and within cultivars remains large. Intraspecific trait variation is a critical consideration to refine regional to global models of agroecosystem structure, function and food security. Considerable opportunities and benefits exist for consolidating a crop trait database for a wider range of domesticated plant species.

## Teaser

Wheat and maize are among the world’s most important crops, covering nearly 400 million ha of Earth’s land surface (an area roughly twice the size of Mexico) and playing a major role in the diets and livelihoods of millions of people. Leaf characteristics (or leaf traits) of these crops—including photosynthetic rates—are widely used in models that predict how wheat and maize will respond to environmental change. Using a large global database, we evaluate how leaf traits of wheat and maize differ across cultivars, and link this variability to climate conditions. In doing so, we assess how leaf traits can be expected to vary under climate change, and in turn, influence predictions of future food security.

## Introduction

Functional traits refer to the structural, chemical, physiological or phenological properties of plants and plant parts, which mechanistically influence plant performance (i.e. growth, survival and reproduction) across environmental gradients (Violle *et al.* 2007). Research on functional traits has been critical in advancing our understanding of the structure and function of terrestrial ecosystems (Reich *et al.* 1997; Westoby and Wright 2006; Díaz *et al.* 2016). Based on a growing number of studies from experimental and natural systems, ecologists have developed a deeper understanding of the key traits that mechanistically underpin plant responses to environmental change. In terrestrial ecology, considerable efforts have focused on identifying the leaf (e.g. Reich *et al.* 1999; Wright *et al.* 2004), root (e.g. Craine *et al.* 2005), reproductive (e.g. Moles *et al.* 2004) and whole-plant traits (e.g. Westoby 1998; Díaz *et al.* 2016) that individually or cumulatively contribute to ecologically important differences in functional biology among species.

Of these groups of traits, leaf functional traits have arguably received the most attention by ecologists and plant ecophysiologists. In particular, the ‘Leaf Economics Spectrum’ (LES) has been hypothesized and tested, as a suite of covarying leaf traits that can be used to describe plant functional biology (Reich *et al.* 1999; Wright *et al.* 2004; Wright *et al.* 2005a). On one end of the LES are ‘resource conserving species’ that express low specific leaf area (SLA), low leaf nitrogen (N) concentrations and low maximum photosynthetic rates (*A*_max_). At the opposite end of the LES are ‘resource acquisitive species’ that express high SLA, high leaf N and high *A*_max_ (Wright *et al.* 2004; Wright *et al.* 2005a). Since publication of hypotheses on the factors governing leaf-level trade-offs—broadly categorized as selection vs. constraints (Lambers and Poorter 1992; Reich *et al.* 1992; Grime *et al.* 1997)—considerable evidence for the existence of a universal LES across plant species worldwide, including both C_3_ and C_4_ plants, has emerged (e.g. Wright *et al.* 2004; Díaz *et al.* 2016). In turn, LES traits now factor heavily into applied research on multiple ecosystem functions including global net primary productivity (e.g. Van Bodegom *et al.* 2012), plant decomposition (e.g. Cornwell *et al.* 2008), disturbance recovery (e.g. Saura-Mas *et al.* 2009), species invasions (e.g. Penuelas *et al.* 2010) and species coexistence (e.g. Kraft *et al.* 2008).

While much contemporary research on plant functional traits has focused on ‘natural ecosystems’, trait-based research also has clear application in agricultural systems (Martin and Isaac 2015; Milla *et al.* 2015; Wood *et al.* 2015; Martin and Isaac 2018). For example, the world’s 65 most common crops occupy ~1.2 billion ha or 8.1 % of the Earth’s land surface (Martin and Isaac 2015). Yet despite these same crop species spanning a continuum of growth forms and strategies, from small-statured fast-growing annuals to slower-growing perennial trees, they commonly remain represented in global dynamic vegetation models as a small number of generalized plant functional types (PFTs) (Monfreda *et al.* 2008). Additionally, LES traits, namely SLA and *A*_max_, are key inputs in many of the world’s most commonly employed crop yield simulation models (e.g. Jones *et al.* 2003; Bouman and van Laar 2006), including those underpinning yield assessments by the Intergovernmental Panel on Climate Change (Porter *et al.* 2014 as summarized here in **Supporting Information—Table S1**). Although crop growth models are parameterized with functional trait data, crops are commonly represented by species-level mean trait values. Accounting for intraspecific variation in LES traits for crops has been identified as a key avenue for refining predictions of agricultural yield (Bouman and van Laar 2006). More broadly then, quantifying intraspecific variation in LES traits for just two crop groups—wheat (*Triticum* spp.) and maize (*Zea mays*)—could aid in refining simulation models of food, nutrient, water and energy fluxes across nearly 400 million ha of crop land (**see Supporting Information—Table S1** in Martin and Isaac 2015).

Recent analyses have pointed to the importance of intraspecific trait variation (ITV) in influencing structure and function in natural or experimental ecosystems (e.g. Albert *et al.* 2010; Kattge *et al.* 2011; Siefert *et al.* 2015). There is also reason to expect ITV is critical in governing agroecological processes. Despite crop cultivars being the result of extensive artificial selection for certain traits, studies have shown that the range of ITV within and among cultivars can be both remarkably wide (e.g. Driever *et al.* 2014), and systematically predicted by certain environmental or management characteristics such as soil nutrient-, water- or light regimes (Donovan *et al.* 2014; Gagliardi *et al.* 2015; Martin *et al.* 2017). In turn, ITV across managed environmental gradients contributes to differences in multiple agroecosystem functioning including plant yield (Bouman and van Laar 2006; Gagliardi *et al.* 2015), biomass accumulation and light interception (Milla *et al.* 2014), and litter decomposition and nutrient cycling (He *et al.* 2012; García-Palacios *et al.* 2013).

Analyses of ITV in LES traits of crops have also been employed to develop and test novel hypotheses on the ecological implications of artificial selection (Milla *et al.* 2015; Martin *et al.* 2017). Authors have hypothesized that artificial selection, coupled with high-resource conditions in agroecosystems, will shift crop traits towards to ‘resource acquiring’ end of the LES, and that such shifts have systematic impacts on rates of agroecosystem functions (Milla *et al.* 2014; Milla *et al.* 2015). Although there is qualitative evidence to support this hypothesis using species average trait values (e.g. **Supporting Information—Fig. S1** in Martin and Isaac 2015; Milla *et al.* 2015), quantitative analyses that define ITV in crops along key axes of the LES could provide more robust support for this expectation (Donovan *et al.* 2014; Martin *et al.* 2017).

Here, we employ the world’s largest functional trait database—the TRY database (Kattge *et al.* 2011)—coupled with an extensive literature review, in order to understand interspecific differences and ITV in wheat and maize, the world’s two most common crops. We specifically focus on ITV in LES traits in these crops, in order to address the following questions: (i) What is the extent of intraspecific variation in LES traits for the world’s most common crops? (ii) What climatic or management-related variables best account for ITV in crops? (iii) Have wheat and maize been shifted towards the extreme resource acquiring end of the LES?

## Methods

### Study species and leaf trait compilation

Our analysis focused on two species of wheat—*Triticum aestivum* (Poaceae), *T. durum*—and *Z. mays* (Poaceae). Focusing on *T*. *aestivum*, *T*. *durum* and *Z*. *mays* is consistent with species-level taxonomy for the ‘Maize’ and ‘Wheat’ commodity groups recognized by the Food and Agricultural Organization of the United Nations (FAO) (see www.fao.org/economic/ess/ess-standards/commodity). (Three additional wheat species, *T*. *dicoccon*, *T*. *monococcum* and *T*. *spelta*, were also initially included in our search, but these species yielded prohibitively low returns (i.e. no data available in the TRY database (see below), and less than three peer-reviewed publications with trait data for each species).) We initially sought to assess ITV at the within-cultivar level. However, trait observations for any particular cultivar across multiple studies were prohibitively low to allow comprehensive assessments of ITV within any cultivar, beyond the results reported in a given source publication (although cultivar identity was reported and accounted for in our analysis here).

For *T*. *aestivum*, *T*. *durum* and *Z*. *mays*, we focused our data compilation on key LES traits that are important inputs into many agricultural vulnerability models, particularly those employed by the IPCC (Porter *et al.* 2014) (reviewed here in **Supporting Information—Table S1**). Data compilation was done following two approaches. First, we submitted structured data requests to the TRY database (Kattge *et al.* 2011). We specifically requested any data records that included information on LES traits including SLA, photosynthesis on an area basis (*A*_max_) and leaf N on both a mass and area basis (Wright *et al.* 2004). We also requested data for additional traits, namely leaf photosynthesis and respiration on mass basis (*A*_mass_ and *R*_d_, respectively), leaf area, leaf phosphorus concentrations, leaf lifespan and leaf dry mass, but there were few data available on these traits, so they were not included in our analysis here.

Second, we complemented the TRY data requests with a structured literature search using the Web of Science journal database and Boolean operators. Specifically, we searched peer-reviewed literature for the terms ‘leaf’ and ‘trait*’, coupled with species names (e.g. ‘leaf nitrogen’ AND ‘*Zea mays*’). Our search targeted (but was not limited to) the period of 2000–15 in order to focus our analysis on the most recent wheat and maize genotypes, the majority of which have been introduced since 2000 (Driever *et al.* 2014) and differ broadly in their traits as compared to earlier genotypes (Roucou *et al.* 2018). We also searched for species names coupled with the physiological terms (e.g. ‘wheat’ AND ‘photosynthesis’). The full text of each article was then searched for these terms. We then limited these studies to include field-based, greenhouse and growth chamber experiments, and where sampling protocols in the studies followed (at least approximately) standardized functional trait collection methodologies (Perez-Harguindeguy *et al.* 2013). Any deviations from these protocols were noted for analysis.

### Environmental data

In both approaches, we sought to obtain any ancillary metadata including spatial location and environmental conditions for all trait data. For trait data found through the literature search, this information was largely derived from the published article. This included geographical information for each trait value (region, country, latitude, longitude, altitude), as well as climate data including mean annual temperature (MAT) and total annual precipitation (TAP). We also sought to include information on solar irradiance, vapour pressure deficit (VPD) and potential evapotranspiration (PET), but these variables were not consistently reported among studies and were therefore not incorporated into our formal analysis. Where climate information was not included in these studies, historical climate data were obtained via the closest weather station, and using a 0.6 °C change in growth temperature (GT), per 100 m in elevation (following Wright *et al.* 2005b). Also, many studies included data on mean GT (the average temperature of the growing season months) as opposed to MAT. Therefore, for all observations GT was calculated using weather station data, averaging all monthly temperatures ≥ 5 °C, following Wright *et al.* (2005b). If published articles reported mean trait values taken over two or more growing seasons, the climatic variables were averaged across the same time frame. In the case of TRY-derived data, when location and/or climatic variables were not indicated in the data releases, individual researchers were contacted to provide these data.

We also sought to record any relevant site-specific data including soil classification, prior land use, monoculture or polyculture management systems, other crop species present, nitrogen (N) fixing plants present, crop planting density, soil pH, N fertilizer application rate, irrigation rates, plant age and day of sampling. Although explicit efforts were made to standardize these data for analysis, since they were not consistently reported among studies (or at all in many analyses), and were challenging/impossible to standardize in certain instances (such as fertilization treatments which were reported in multiple inconsistent ways), they were generally omitted from analyses here (but see below).

All trait measurements were additionally categorized by study area type, which was listed as one of ‘field’, ‘greenhouse’ or ‘growth chamber’. Any pot experiments within the compilation were categorized as either field or greenhouse, depending on the conditions of the experiment. If both field and greenhouse locations were used within one study, the data were separated when possible. Other ancillary information included the plant developmental stage at time of sampling. Due to inconsistent methods of reporting across studies, we sought to re-categorize this information for analysis. For example, any one of the following was commonly given: date of sowing; date of sampling; number of days after a given development stage; or the plant developmental stage at time of sampling. This reporting also included varying developmental stage models (e.g. Zadoks 1985; Lancashire *et al.* 1991). Developmental stages were therefore converted into the most common measurement found in our study: days since planting (D). For maize, developmental stages were converted into D using ranges of the number of days that is typically required for each growth stage. For wheat, developmental stages within each study were converted to D following a generalization from Dias *et al.* (2011) (i.e. the number of days after emergence required for each growth stage) taking into account an additional 10-day period for sowing to emergence.

### Functional trait data availability

**Figure 1.**
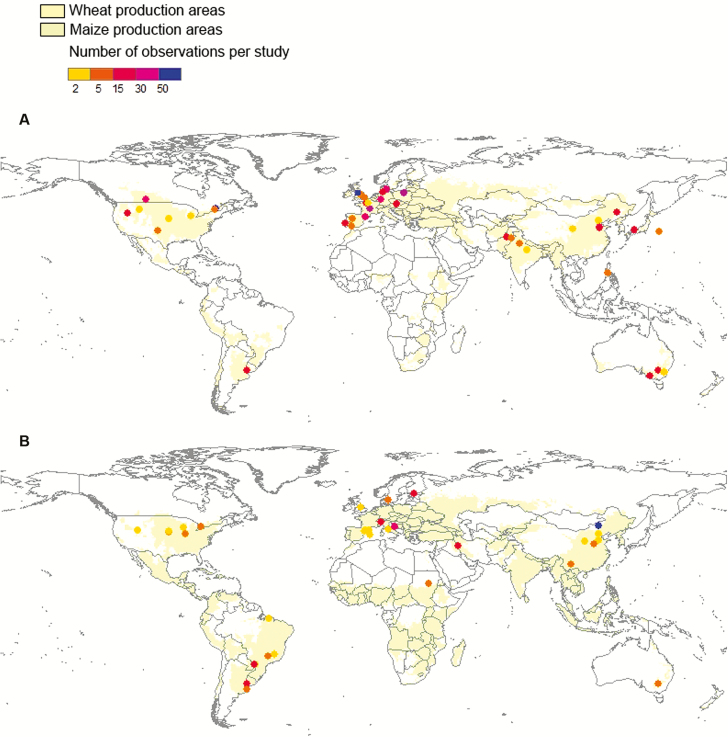
Leaf trait observations for *Triticum aestivum* and *Triticum durum* (panel A) and *Zea mays* (panel B), as compared to the growing regions for both crop groups (according to Monfreda *et al.* 2008). Colours correspond to the number of observations available from each study location.

Our data compilation resulted in a total of 721 leaf level observations for *Z*. *mays*, *T*. *aestivum* and *T*. *durum* taken from 75 studies conducted across 23 countries and 67 different regions (Fig. 1; **see Supporting Information—Table S2**). Trait observations were more readily available in the primary literature (*n* = 663 observations) as compared to the TRY database (*n* = 122 observations), and the large majority of data were based on field-grown crops (*n* = 564 observations across 44 studies, of which *n* = 61 observations from eight studies were based on potted plants), as compared to growth chambers (*n* = 168 observations from 16 studies) or greenhouse-based studies (*n* = 45 observations from nine studies); one study entailed *n* = 2 observations, one from both field- and greenhouse conditions, while *n* = 8 observations from five studies did not provide growth condition information.

Geographic representation of crop trait data varied with the largest number of sources derived from studies based in Europe (*n* = 292 observations across 31 studies) and Asia (*n* = 136 observations across 21 studies), followed by Australia (*n* = 95 observations across four studies), South America (*n* = 32 observations across four studies) and North America (*n* = 136 observations across four studies); Africa was represented by *n* = 4 observations, and *n* = 1 observation from one study was not associated with any geographical information (Fig. 1; **see Supporting Information—Table S2**).

### Data analysis—trait variation among and within crop species

All statistical analyses were conducted using R version 3.3.0 (R Core Team 2016). For each individual trait we calculated descriptive statistics for each species individually, and tested for differences among species. Sample sizes for each trait-by-species combination were unbalanced, and different data sources contributed to our data set unequally. Therefore, descriptive statistics were based on linear mixed-effects models performed using the ‘nlme’ R package (Pinheiro *et al.* 2016), where species identity was treated as a fixed factor and genotype as a random factor. Based on these models, we calculated least squares mean values and associated SEs for each trait on a species-by-species basis using the ‘lsmeans’ R package (Lenth 2016). To quantify any systematic bias in traits as a result of genotype, for each model we also used the ‘piecewiseSEM’ package (Lefcheck 2016) to calculate both marginal *r*^2^ and conditional *r*^2^ values, which represent the variance in a given trait explained by the fixed factors alone (i.e. species identity) and the variance explained by both the fixed and random factors (i.e. species identity and genotype, respectively) (Nakagawa and Schielzeth 2013).

To understand where wheat and maize fell within the LES, we compared mean crop trait values to the trait variation observed in the GLOPNET database, which is the initial global data set (comprised of both C_3_ and C_4_ plants) used to define the LES (Wright *et al.* 2004). Specifically, this was done by comparing mean crop trait values, as well as the range of ITV in crop traits, to quantiles calculated for SLA, *A*_max_ and leaf N across the entire GLOPNET data set (where *n* = 2370 for SLA, *n* = 764 for *A*_max_ and *n* = 2061 for leaf N).

### Data analysis—trait–environment relationships

To evaluate the influence of temperature and precipitation on leaf traits, we followed a multi-step process. First, we fit a preliminary mixed-effects model for each trait individually pooled across all species, in order to understand how other environmental or management-related variables (largely measured as binary or categorical variables) might confound relationships between traits and GT and TAP. To do so, we fit a model of the form:

$$trait_{i}=\beta_{0}+\beta_{1}N_{i}+\beta_{2}I_{i}+\beta_{3}F_{i}+\beta_{4}D_{i}+(G_{i}+\varepsilon_{i})$$ (1)

where trait_*i*_ represents the value of the *i*th trait measurement in our data set, which is predicted as a function of five fixed effects including (i) *β*_0_, which represents an overall model intercept; (ii) *β*_1_, which is the parameter estimate associated with nitrogen fertilization (N, treated here as a binary variable); (iii) *β*_2_, which is the parameter estimate for irrigation (I, treated as a binary variable); (iv) *β*_3_, which is the parameter estimate for the type of study (F: field, greenhouse or growth chamber); and (v) *β*_4_, which is the parameter estimate that represents the influence of days since planting (D). In this model, the influence of crop genotype (G_*i*_) on a predicted trait value was included as a random effect, and *ε*_*i*_ represents the error associated with each individual trait observation.

Based on the results of these analyses **[see Supporting Information—Table S3]**, in our second step we fit and compared a number of linear mixed-effects models to evaluate relationships between traits and both GT and TAP. These full models included species identity, GT, TAP and all species-by-environment interactions as fixed effects, and variables that had a significant influence on traits (as per Equation 1 and **Supporting Information—Table S3**) as random effects. These full models were of the form:

$$A_{max-ij}=\beta_{0}+\beta_{1}S_{j}+\beta_{2}GT_{ij}+\beta_{3}TAP_{ij}+\beta_{4}S_{j}GT_{ij}+\beta_{5}S_{j}TAP_{ij}+(D_{ij}+\varepsilon_{ij})$$ (2)

$$Leaf\;N_{ij}=\beta_{0}+\beta_{1}S_{j}+\beta_{2}GT_{ij}+\beta_{3}TAP_{ij}+\beta_{4}S_{k}GT_{ij}+\beta_{5}S_{k}TAP_{ij}+(N_{ij}+\varepsilon_{ij})$$ (3)

$$SLA_{ij}=\beta_{0}+\beta_{1}S_{j}+\beta_{2}GT_{ij}+\beta_{3}TAP_{ij}+\beta_{4}S_{k}GT_{ij}+\beta_{5}S_{k}\text{TA}P_{ij}+(I_{ij}+F_{ij}+D_{ij}+\varepsilon_{ij})$$ (4)

where trait_*ij*_ represents the predicted trait value measured on the *i*th leaf of the *j*th species, and *β*_0_ represents an overall intercept. In these models, (i) *β*_1_ represents the parameter estimate associated with the *j*th species (S, which in these analysis represents the parameter estimate associated with *Z*. *mays* only; since *T*. *durum* trait observations did not have associated climate information, only two species are addressed here); (ii) *β*_2_ is the parameter estimate for GT; (iii) *β*_3_ represents the parameter estimate for TAP; (iv) *β*_4_ represents an interaction term between S and GT (a parameter applicable only to *Z*. *mays*); and (v) *β*_5_ is the interaction term between S and TAP (a parameter also applicable only to *Z*. *mays*). Random effects in these models (included according to significant parameters detected in the analyses at Equation 1 and **Supporting Information—Table S3**) were irrigation (I_*ij*_), the type of study (F_*ij*_), nitrogen fertilization (N_*ij*_) and the number of days since planting (D_*ij*_) on predicted trait values; *ε*_*ij*_ represents the error associated with each individual trait observation. Sample sizes of these models (i.e. where trait values were paired with all of the fixed and random effects noted in Equations 2–4) were *n* = 133 for *A*_max_, *n* = 206 for leaf N and *n* = 34 for SLA.

In our next analysis step we used Akaike information criterion (AIC) scores to compare the full models in Equations 2–4 to reduced models that included different combinations of fixed effects (specified in **Supporting Information—Table S4**). In general, the full models presented were associated with either the lowest AIC score, or had AIC scores that were ≤2 greater than the AIC scores for the next most parsimonious model fit **[see Supporting Information—Table S4]**. Therefore, in our next step we assessed the statistical significance of all fixed effects in Equations 2–4, in order to inform our final predictive model. Specifically, for each model predicting trait values, all non-significant fixed effects were removed **[see Supporting Information—Table S5]**, leaving us with final predictive models of each individual trait (Table 2). For each of the final predictive models we also calculated both marginal *r*^2^ and conditional *r*^2^ values.

## Results

Across crop species, *T. aestivum* had the most extensive LES trait coverage with *n* = 496 observations, while *Z. mays* traits were represented by *n* = 207 observations; *T. durum* was represented by only *n* = 18 observations. For *T. aestivum*, leaf N concentration was the most well-represented trait (*n* = 218), followed by *A*_max_ (*n* = 173) and SLA (*n* = 105) (Table 1). Similarly for *Z. mays*, leaf N was the most commonly observed leaf trait (*n* = 88), followed by *A*_max_ (*n* = 80) and SLA (*n* = 39). Sample sizes for *T*. *durum* were considerably lower for *A*_max_ (*n* = 9), leaf N (*n* = 8) and SLA (*n* = 1) (Table 1).

**Table 1.** Sample sizes and descriptive statistics for three leaf-level functional traits for wheat (*Triticum aestivum* and *Triticum durum*) and maize (*Zea mays*). Marginal means are derived from a linear mixed-effects model predicting trait values as a function of species, while accounting for potential systematic bias associated with unequal sample sizes across genotypes. In addition to observed ranges, IQRs are also provided for each trait. Explained variance for a given trait is presented as the proportion explained by species identity alone (marginal *r*^2^), and the proportion explained by species identity and genotype (conditional *r*^2^). Units are as follows: SLA, mm^2^ mg^−1^; leaf N, mg g^−1^; *A*_max_, µmol CO_2_ m^−2^ s^−1^.

Trait	*T. aestivum*			*T. durum*			*Z. mays*			Explained variance	
	*n*	Marginal mean (SE)	Observed range (IQR)	*n*	Marginal mean (SE)	Observed range (IQR)	*n*	Marginal mean (SE)	Observed range (IQR)	Marginal *r*^2^	Conditional *r*^2^
*A*_max_	237	23.1 (0.8)	2–39 (16.0–28.0)	9	24.4 (3.4)	11.5–29.8 (17.5–29.0)	80	27.4 (1.7)	12.8–47.3 (17.0–28.2)	0.294	0.64
Leaf N	218	34.2 (0.8)	6.1–58.7 (26.5–42.2)	8	36.1 (3.0)	22.1–46.0 (33.8–40.3)	88	30.7 (1.1)	13.6–70.7 (23.8–36.0)	0.026	0.572
SLA	105	20.6 (1.1)	7.5–44.7 (15.1–29.6)	1	9.6 (NA)	NA (NA)	39	22.6 (1.6)	10.0–36.4 (16.0–28.2)	0.034	0.678

### Inter- and intraspecific trait variation

**Figure 2.**
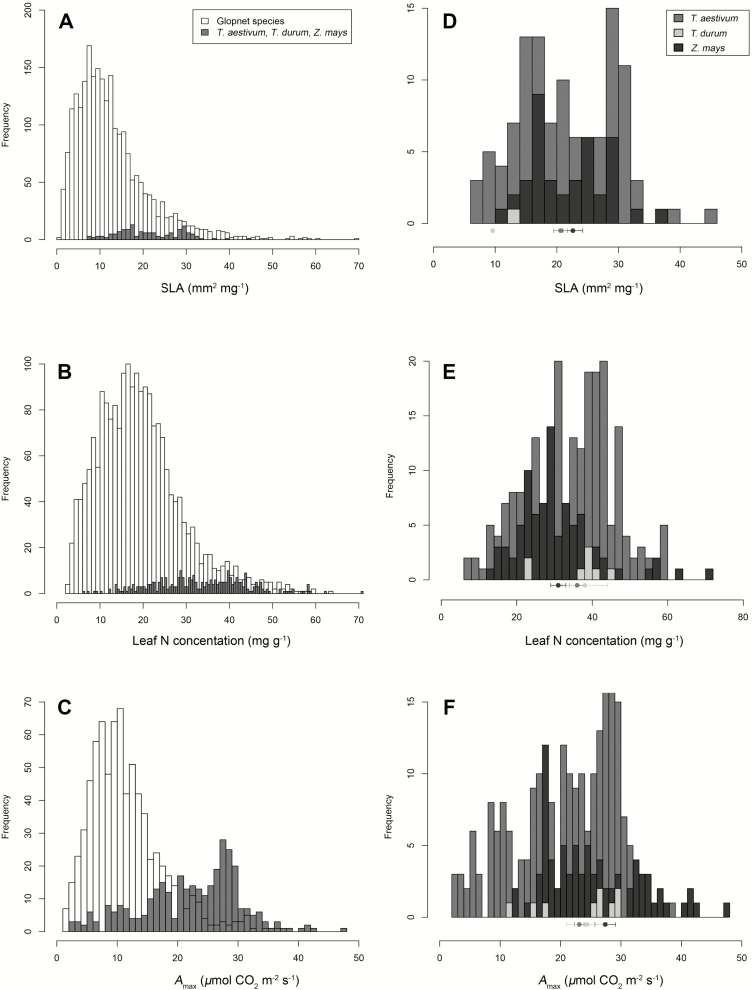
Intraspecific variation in leaf functional traits for *Triticum aestivum*, *Triticum durum* and *Zea mays* as compared to traits in a global species pool. Panels A–C represent the distribution of all crop trait values (dark grey bars) as compared to the GLOPNET data set (open bars). Panels D–F represent crop species distributions, and points below the histograms correspond to species least square mean values (see Table 1) with error bars corresponding to ±1 SE of the mean.

**Figure 3.**
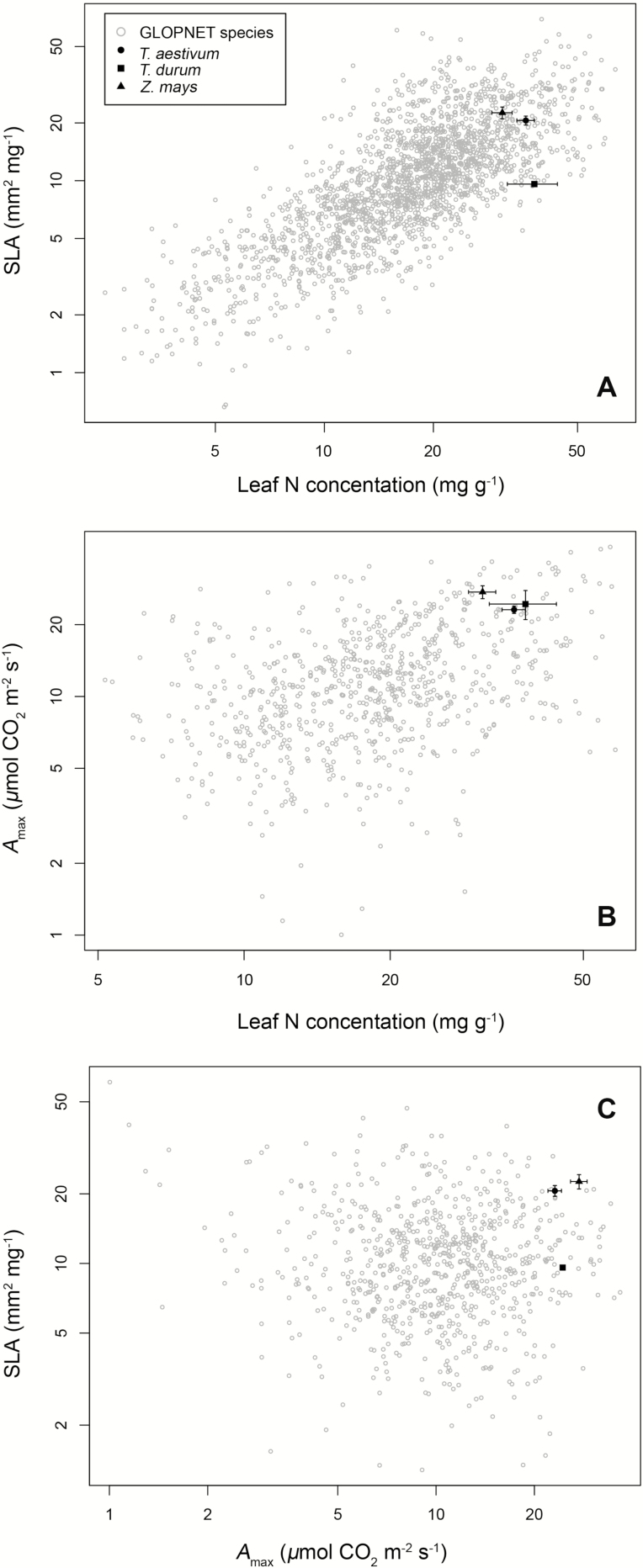
Intraspecific variation in leaf functional traits for *Triticum aestivum*, *Triticum durum* and *Zea mays*. Panels A–C represent three different bivariate trait trade-offs along the Leaf Economics Spectrum. Filled black symbols correspond to species-specific least square mean trait values with error bars corresponding to ±1 SE of the mean (see Table 1). For comparison, all species in the GLOPNET data set (open gray circles) are also shown.

Mean *A*_max_ was highest in *Z. mays* (27.4 ± 1.7 (SE) µmol CO_2_ m^−2^ s^−1^), followed by *T. durum* (24.3 ± 3.4 µmol CO_2_ m^−2^ s^−1^) and *T. aestivum* (16.4 ± 1.2 µmol CO_2_ m^−2^ s^−1^), with wheat species differing significantly from maize (Table 1; Fig. 2). Similarly, mean SLA differed significantly across species, ranging from 9.6 mm^2^ mg^−1^ in *T*. *durum*, to 22.6 ± 1.6 mm^2^ mg^−1^ in *Z*. *mays*, to 20.6 ± 1.1 mm^2^ mg^−1^ in *T. aestivum* (Table 1; Figs 2 and 3). Mean leaf N concentrations did not differ significantly among crop species, ranging from 30.7 ± 1.1 mg g^−1^, 34.2 ± 0.8 mg g^−1^ and 36.1 ± 3.0 mg g^−1^ in *Z. mays*, *T. aestivum* and *T*. *durum*, respectively (Table 1; Figs 2 and 3). Compared to interspecific variation, ITV was large. Specifically, SLA varied over 3-fold in *Z*. *mays* (range = 10.1–36.4 mm^2^ mg^−1^, interquartile range (IQR) = 16.0–28.8 mm^2^ mg^−1^) and 6-fold in *T*. *aestivum* (range = 7.5–44.7 mm^2^ mg^−1^, IQR = 15.1–29.6 mm^2^ mg^−1^), while leaf N varied over 2-fold in *T*. *durum* (range = 22.1–46.0 mg g^−1^, IQR = 33.8–40.3 mg g^−1^), 5-fold in *Z*. *mays* (range = 13.6–70.7 mg g^−1^, IQR = 23.8–36.0 mg g^−1^) and nearly 10-fold in *T*. *aestivum* (range = 6.1–58.7 mg g^−1^, IQR = 26.5–42.2 mg g^−1^; Table 1; Fig. 2). Similarly, *A*_max_ varied nearly 3-fold in *Z*. *mays* (range = 12.8–47.3 µmol CO_2_ m^−2^ s^−1^, IQR = 17.0–28.5 µmol CO_2_ m^−2^ s^−1^), 19-fold in *T*. *aestivum* (range = 2.0–39.0 µmol CO_2_ m^−2^ s^−1^, IQR = 16.0–28.0 µmol CO_2_ m^−2^ s^−1^) and 2-fold in *T*. *durum* (range = 11.5–29.8 µmol CO_2_ m^−2^ s^−1^, IQR = 17.5–29.0 µmol CO_2_ m^−2^ s^−1^; Table 1; Fig. 2).

As compared to a global species pool, mean wheat and maize trait values fell within the upper 95th percentile of all three key LES traits examined here. Specifically, as compared to the GLOPNET species pool, mean *A*_max_ values for all three crops fell between the 95th and 99th percentile of values (which ranged in GLOPNET from 22.8 to 31.4 µmol CO_2_ m^−2^ s^−1^), and leaf N values fell within the 90th and 99th percentile (which ranged in GLOPNET from 31.9 to 50.5 mg g^−1^). Mean SLA values for *T*. *aestivum* and maize fell within the 85th percentile (which ranged in GLOPNET from 20.5 to 24.1 mm^2^ mg^−1^), although mean SLA for *T*. *durum* fell only within the 40th percentile of GLOPNET values (Figs 2 and 3).

### Correlates of ITV

Our preliminary analysis found that the number of days since planting had a significant influence on *A*_max_**[see Supporting Information—Table S3]**. When accounting for these factors, species identity, GT, TAP and a species-by-GT interaction term explained 35.3 % of the variation in *A*_max_ (Table 2). This relationship includes a significant positive relationship between TAP and *A*_max_, a significant positive relationship between GT and *A*_max_ in *T*. *aestivum* (Table 2) and a negative relationship between GT and *A*_max_ in *Z*. *mays* that was significantly different from the relationship found in *T*. *aestivum* (Table 2). An additional 35.6 % of the variation in *A*_max_ was attributable to days since sowing (Table 2).

**Table 2.** Variation in three functional traits in relation to GT and precipitation. For each trait, only significant fixed effects were incorporated (based on the results of an AIC model comparison (**see Supporting Information—Tables S4 and** **S5**)), which included species identity (S), GT, TAP and associated interaction terms (denotes by ‘*’). In these models, *Zea mays* was coded as a dummy variable in order to evaluate differences among species (S). Therefore, any parameters that include ‘S’ are associated with *Z*. *mays* only. Significant model parameters (where *P* ≤ 0.05) are highlighted in bold. Random effects were also included in these models based on preliminary analysis (as per Equation 1 and **Supporting Information—Table S3**). Specifically, across the three different models random effects included (i) the number of days since planting for *A*_max_, (ii) nitrogen fertilization only for leaf N and (iii) the number of days since planting, irrigation and type of study for SLA.

Trait	Parameter	Estimate	SE	d.f.	*t*-value	*P*-value
*A*_max_ (*n* = 197)	**Intercept**	−**13.41**	**6.49**	**161**	−**2.07**	**0.0404**
	**S**	**48.89**	**11.26**	**161**	**4.34**	**<0.001**
	**GT**	**0.93**	**0.38**	**161**	**2.43**	**0.0162**
	**TAP**	**0.02423**	**0.003**	**161**	**7.93**	**<0.001**
	S * GT	−**1.82035**	**0.7**	**161**	−**2.61**	**0.01**
	S * TAP	−**0.01**	**0.01**	**161**	−**2.298**	**0.023**
	Model marg. *r*^2^	0.394				
	Model cond. *r*^2^	0.73				
Leaf N (*n* = 206)	**Intercept**	**41.7**	**5.8**	**201**	**7.16**	**<0.001**
	**GT**	−**0.9**	**0.2**	**201**	−**3.97**	**0.0001**
	**TAP**	**0.01**	**0.003**	**201**	**2.33**	**0.021**
	S * GT	−0.04	0.1	201	−0.47	0.642
	Model marg. *r*^2^	0.054				
	Model cond. *r*^2^	0.355				
SLA (*n* = 34)	**Intercept**	**46.63**	**8.48**	**21**	**5.5**	**<0.001**
	**GT**	−**1.33**	**0.42**	**21**	−**3.19**	**0.004**
	**TAP**	−**0.014**	**0.003**	**21**	−**4.36**	**0.0003**
	Model marg. *r*^2^	0.437				
	Model cond. *r*^2^	0.966				

Growth temperature and TAP alone explained 43.7 % of the variation in SLA, with both of these environmental variables being significant negative predictors of this trait (Table 2). These patterns did not differ significantly across species (Table 2). Generally, for a 1 °C increase in temperature, SLA declined by 1.3 ± 0.4 mm^2^ mg^−1^ (SE), while for every 1 mm increase in precipitation SLA declined by 0.02 ± 0.003 mm^2^ mg^−1^ (Table 2). Time since sowing, the presence of absence of irrigation and the type of study explained an additional 52.9 % of the variation in SLA, with especially notable declines in SLA occurring as the number of days since planting increased **[see Supporting Information—Fig. S3]**.

Growth temperature was significantly negatively related to leaf N in both species, with a 1 °C increase in temperature associated with a 0.9 ± 0.2 mg g^−1^ decline in leaf N (Table 2). This pattern did not differed significantly across species (Table 2). Leaf N concentrations were also significantly related with TAP, but these relationships were weak (Table 2). Overall, climatic effects only explained 5.4 % of the variation in leaf N, while fertilization status (included as a random effect) explained an additional 30.1 % of the variation (Table 2).

## Discussion

### Trait–environment relationships

Research in natural systems has placed considerable effort on understanding relationships between interspecific trait variation and environmental conditions. This literature has generally reported systematic covariation between traits and environment (e.g. Craine *et al.* 2005; Wright *et al.* 2005b; Reich *et al.* 2007; Ordoñez *et al.* 2009; Maire *et al.* 2015). Among traits examined here our results indicate ITV in SLA and *A*_max_, but not leaf N, was most strongly linked to climate (Table 2). Specific leaf area and *A*_max_ are key inputs into the world’s most prominent crop yield simulation models **[see Supporting Information—Table S1]**. Based on our results, accounting for variation in these traits across environmental gradients is key in refining predictions and projections of agricultural yield.

Here we observed a pattern of declining SLA with increasing temperature, which was consistent between both *T*. *aestivum* and *Z*. *mays* (Table 2). Studies have linked declines in SLA, or alternatively increases in leaf mass per area (LMA), with increasing temperature as a function of changes in evaporative demand during leaf expansion. Specifically, under higher temperatures where evaporative demand is expected to be higher, mesophyll cell, lignin and phenolic compounds are generally expressed in greater concentrations, leading to leaves with lower SLA (Poorter *et al.* 2009). Although growth irradiance clearly plays an additional key role in moderating SLA expression within species (Lusk *et al.* 2008), our findings are consistent with literature suggesting that higher temperatures and high evaporative demand drive declines in SLA. In *T*. *aestivum* we observed a positive relationship between *A*_max_ and GT, which is consistent with the ability of photosynthesis in C_3_ plants to acclimate to shifts in thermal regimes (Hikosaka *et al.* 2006). More specifically, the literature on limits to photosynthesis in C_3_ plants indicates that the positive GT–*A*_max_ trend in *T*. *aestivum* could be related to plants overcoming RuBP regeneration as temperatures increase (Hikosaka *et al.* 2006).

From a modelling perspective, studies have suggested that accounting for intraspecific variation in SLA and/or *A*_max_ that occurs across sites is a key data consideration when refining models that predict crop yield (Bouman and van Laar 2006). Our results here more specifically indicate that accounting for ITV in wheat and maize among sites that differ in temperature and precipitation is more critical in capturing ITV in these traits, as compared to differences among soil management regimes (Table 2; **see Supporting Information—Table S3; Figs S1 and S2**).

### Extent of ITV in wheat and maize

Although the past 15–20 years have seen a remarkable increase in functional trait-based research for wild plants in terrestrial ecosystems, many of these same traits have been of keen interest to agronomists and crop physiologists for decades. But to date, data from such studies remain unconsolidated. Our results confirm the presence of hundreds of observations of *A*_max_, SLA and leaf N, which highlights systematic differences among these traits in the world’s two most widespread crops (Table 1).

Our data indicate that wheat and maize traits span a wide breadth of the LES that has been observed in wild plant species (Fig. 2), suggesting that ITV should be recognized and incorporated into analyses of agroecosystem function that rely on these traits **[see Supporting Information—Table S1]**. However, these data should be interpreted or employed carefully. Specifically, process-based models of crop yield are interested mainly in modelling crop physiological rates at certain ontogenetic stages, such as post-grain-filling or other stages of reproductive development. But our data set includes leaves across a range of ontogenetic stages and environmental conditions, which would not necessarily apply to all wheat or maize plants. Considering the prevalence of SLA and *A*_max_ as a model input (**see Supporting Information—Table S1**; Jones *et al.* 2003; Bouman and van Laar 2006), and the strong variation in these traits across plant ontogeny and environment (Table 2; **see Supporting Information—Fig. S3**), an understanding of the linkages between ITV in LES traits and environmental conditions should be taken into account when parameterizing process-based models with trait data. Our compiled data set (available in the Dryad data repository: doi:10.5061/dryad.4r55n) could be used as a direct source of *A*_max_ and SLA values for crop model parameterization, since these data are linked to specific environmental conditions (GT and TAP) and plant ontogenetic stages (i.e. time since sowing). Similarly, our regression models on trait–environment relationships (Table 2) could also be used to estimate *A*_max_ or SLA values under different environmental conditions, particularly in instances where site-specific trait data are unavailable.

While our data compilation initially indicates extensive coverage of LES traits for wheat and maize, these numbers may actually still not do justice to the environmental and socio-economic importance and extent of these crops globally. Current data from the Food and Agricultural Organization of the United Nations (faostat.fao.org) suggest that wheat and maize occupies ~215 and 120 million ha of cropland, respectively, distributed widely across the globe (Fig. 1) (Martin and Isaac 2015). Based on the number of trait observations (Table 1), and assuming that these observations were perfectly distributed spatially across the growing regions of wheat and maize, this equates to one observation of *A*_max_ across roughly every 1.1 million ha of wheat cropland and one *A*_max_ value for roughly every 1.4 million ha of maize cropland. The highest sample sizes in our data set were for leaf N in wheat, which would equate to approximately one leaf N value for every 950000 ha of wheat cropland.

Crop trait compilations could be expanded through data provided by agronomic institutions (Martin and Isaac 2015). Although this has proven useful for enhancing meta-analyses of traits strictly associated with crop yield or other aspects of domestication (Meyer *et al.* 2012), it has to date been less effective for other crop traits such as those comprising the LES (Martin and Isaac 2015). Indeed, our data compilation efforts did not benefit from data available from agronomic institutions, including the International Maize and Wheat Improvement Centre (www.cimmyt.org). Navigating the landscape of publically funded or proprietary crop data from agronomic institutions may be an avenue for expanding crop trait databases.

## Conclusions

Small-scale, regional- or site-specific vulnerability assessments of crop growth and yield are gaining importance in the assessment of agroecosystem structure and function, with a growing recognition that large-scale models can be broadly informative but limited in terms of supporting management decisions at a farm scale. Advances in techniques for downscaling climate models now allow for high-resolution climate change projections at fine spatial scales. But even so, there remain systematic deficiencies in even the most computationally intensive models (i.e. regional climate models coupled with crop simulators) to reproduce observed yields (Glotter *et al.* 2014).

The incorporation of coupled trait–environment data into these analyses is a tractable way to refine fine-scale models of crop growth and yield, but to date, such data have not been readily available (Bouman and van Laar 2006). As a result, researchers commonly rely on previously obtained data on trait–environment relationships that are broadly generalized across crop varieties and species. More specifically, trait–environment relationships commonly remain static throughout model assessments, or are otherwise commonly represented as mean species-specific traits under a particular set of environmental conditions (e.g. Jones *et al.* 2003; Bouman and van Laar 2006).

As food security continues to emerge as one of the defining challenges of contemporary climate science, understanding crop responses to climate change remains a critical avenue of research in agroecology. Our analysis suggests that principles and methods commonly employed in functional trait-based ecology can also contribute to these goals. Specifically, global evaluations of inter- and intraspecific variation in crop traits, coupled with testing hypotheses on how functional traits covary within crops, can contribute both basic and applied information that is critical for understanding the structure, function and management of agroecosystems globally.

## Sources of Funding

The authors were supported by the Canada Research Chairs programme and M. E. Isaac, which funded a graduate scholarship to C.E.H. The TRY initiative and database is hosted, developed and maintained at the Max-Planck-Institute for Biogeochemistry (MPI-BGC) in Jena, Germany. TRY is or has been supported by DIVERSITAS, IGBP, the Global Land Project, the UK Natural Environment Research Council (NERC) through its programme QUEST (Quantifying and Understanding the Earth System), the French Foundation for Biodiversity Research (FRB) and GIS ‘Climat, Environnement et Société’ France.

## Contributions by the Authors

A.R.M. conceived the project, compiled data, lead data analyses and prepared the manuscript. C.E.H. lead data compilation, contributed to data analysis and helped prepare the manuscript. B.E.L.C. contributed trait data. J.H.C.C. contributed trait data and advised on data analysis. J.C. contributed trait data and advised on data presentation. W.A.G. contributed to climate data analysis. J.K. contributed trait data and facilitated trait data compilation through the TRY Functional Trait Database. C.K.F.T. compiled data and assisted with data analysis. All authors discussed the results, commented on the manuscript and contributed to revisions.

## Conflict of Interest

None declared.

## Supplementary Material

Supporting Information

## References

[CIT0001] Albert CH, Thuiller W, Yoccoz NG, Douzet R, Aubert S, Lavorel S 2010 A multi-trait approach reveals the structure and the relative importance of intra- vs. interspecific variability in plant traits. Functional Ecology 24:1192–1201.

[CIT0002] Bouman BAM, van Laar HH 2006 Description and evaluation of the rice growth model ORYZA2000 under nitrogen-limited conditions. Agricultural Systems 87:249–273.

[CIT0003] Cornwell WK, Cornelissen JH, Amatangelo K, Dorrepaal E, Eviner VT, Godoy O, Hobbie SE, Hoorens B, Kurokawa H, Pérez-Harguindeguy N, Quested HM, Santiago LS, Wardle DA, Wright IJ, Aerts R, Allison SD, van Bodegom P, Brovkin V, Chatain A, Callaghan TV, Díaz S, Garnier E, Gurvich DE, Kazakou E, Klein JA, Read J, Reich PB, Soudzilovskaia NA, Vaieretti MV, Westoby M 2008 Plant species traits are the predominant control on litter decomposition rates within biomes worldwide. Ecology Letters 11:1065–1071. 18627410 10.1111/j.1461-0248.2008.01219.x

[CIT0004] Craine JM, Lee WG, Bond WJ, Williams RJ, Johnson LC 2005 Environmental constraints on a global relationship among leaf and root traits of grasses. Ecology 86:12–19.

[CIT0005] Dias AS, Semedo J, Ramalho JC, Lidon FC 2011 Bread and durum wheat under heat stress: a comparative study on the photosynthetic performance. Journal of Agronomy and Crop Science 197:50–56.

[CIT0006] Díaz S, Kattge J, Cornelissen JH, Wright IJ, Lavorel S, Dray S, Reu B, Kleyer M, Wirth C, Prentice IC, Garnier E, Bönisch G, Westoby M, Poorter H, Reich PB, Moles AT, Dickie J, Gillison AN, Zanne AE, Chave J, Wright SJ, Sheremet’ev SN, Jactel H, Baraloto C, Cerabolini B, Pierce S, Shipley B, Kirkup D, Casanoves F, Joswig JS, Günther A, Falczuk V, Rüger N, Mahecha MD, Gorné LD 2016 The global spectrum of plant form and function. Nature 529:167–171. 26700811 10.1038/nature16489

[CIT0007] Donovan LA, Mason CM, Bowsher AW, Goolsby EW, Ishibashi CDA 2014 Ecological and evolutionary lability of plant traits affecting carbon and nutrient cycling. Journal of Ecology 102:302–314.

[CIT0008] Driever SM, Lawson T, Andralojc PJ, Raines CA, Parry MA 2014 Natural variation in photosynthetic capacity, growth, and yield in 64 field-grown wheat genotypes. Journal of Experimental Botany 65:4959–4973. 24963002 10.1093/jxb/eru253 PMC4144772

[CIT0009] Gagliardi S, Martin AR, Virginio ED, Rapidel B, Isaac ME 2015 Intraspecific leaf economic trait variation partially explains coffee performance across agroforestry management regimes. Agriculture Ecosystems & Environment 200:151–160.

[CIT0010] García-Palacios P, Milla R, Delgado-Baquerizo M, Martín-Robles N, Alvaro-Sánchez M, Wall DH 2013 Side-effects of plant domestication: ecosystem impacts of changes in litter quality. The New Phytologist 198:504–513. 23356416 10.1111/nph.12127

[CIT0011] Glotter M, Elliott J, McInerney D, Best N, Foster I, Moyer EJ 2014 Evaluating the utility of dynamical downscaling in agricultural impacts projections. Proceedings of the National Academy of Sciences of the United States of America 111:8776–8781. 24872455 10.1073/pnas.1314787111 PMC4066535

[CIT0012] Grime JP, Thompson K, Hunt R, Hodgson JG, Cornelissen JHC, Rorison IH, Hendry GAF, Ashenden TW, Askew AP, Band SR, Booth RE, Bossard CC, Campbell BD, Cooper JEL, Davison AW, Gupta PL, Hall W, Hand DW, Hannah MA, Hillier SH, Hodkinson DJ, Jalili A, Liu Z, Mackey JML, Matthews N, Mowforth MA, Neal AM, Reader RJ, Reiling K, RossFraser W, Spencer RE, Sutton F, Tasker DE, Thorpe PC, Whitehouse J 1997 Integrated screening validates primary axes of specialisation in plants. Oikos 79:259–281.

[CIT0013] He WM, Shen Y, Cornelissen JHC 2012 Soil nutrient patchiness and plant genotypes interact on the production potential and decomposition of root and shoot litter: evidence from short-term laboratory experiments with *Triticum aestivum*. Plant and Soil 353:145–154.

[CIT0014] Hikosaka K, Ishikawa K, Borjigidai A, Muller O, Onoda Y 2006 Temperature acclimation of photosynthesis: mechanisms involved in the changes in temperature dependence of photosynthetic rate. Journal of Experimental Botany 57:291–302. 16364948 10.1093/jxb/erj049

[CIT0015] Jones JW, Hoogenboom G, Porter CH, Boote KJ, Batchelor WD, Hunt LA, Wilkens PW, Singh U, Gijsman AJ, Ritchie JT 2003 The DSSAT cropping system model. European Journal of Agronomy 18:235–265.

[CIT0016] Kattge J, Diaz S, Lavorel S, Prentice C, Leadley P, Bonisch G, Garnier E, Westoby M, Reich PB, Wright IJ, Cornelissen JHC, Violle C, Harrison SP, van Bodegom PM, Reichstein M, Enquist BJ, Soudzilovskaia NA, Ackerly DD, Anand M, Atkin O, Bahn M, Baker TR, Baldocchi D, Bekker R, Blanco CC, Blonder B, Bond WJ, Bradstock R, Bunker DE, Casanoves F, Cavender-Bares J, Chambers JQ, Chapin FS, Chave J, Coomes D, Cornwell WK, Craine JM, Dobrin BH, Duarte L, Durka W, Elser J, Esser G, Estiarte M, Fagan WF, Fang J, Fernandez-Mendez F, Fidelis A, Finegan B, Flores O, Ford H, Frank D, Freschet GT, Fyllas NM, Gallagher RV, Green WA, Gutierrez AG, Hickler T, Higgins SI, Hodgson JG, Jalili A, Jansen S, Joly CA, Kerkhoff AJ, Kirkup D, Kitajima K, Kleyer M, Klotz S, Knops JMH, Kramer K, Kuhn I, Kurokawa H, Laughlin D, Lee TD, Leishman M, Lens F, Lenz T, Lewis SL, Lloyd J, Llusia J, Louault F, Ma S, Mahecha MD, Manning P, Massad T, Medlyn BE, Messier J, Moles AT, Muller SC, Nadrowski K, Naeem S, Niinemets U, Nollert S, Nuske A, Ogaya R, Oleksyn J, Onipchenko VG, Onoda Y, Ordonez J, Overbeck G, Ozinga WA, Patino S, Paula S, Pausas JG, Penuelas J, Phillips OL, Pillar V, Poorter H, Poorter L, Poschlod P, Prinzing A, Proulx R, Rammig A, Reinsch S, Reu B, Sack L, Salgado-Negre B, Sardans J, Shiodera S, Shipley B, Siefert A, Sosinski E, Soussana JF, Swaine E, Swenson N, Thompson K, Thornton P, Waldram M, Weiher E, White M, White S, Wright SJ, Yguel B, Zaehle S, Zanne AE, Wirth C 2011 TRY - a global database of plant traits. Global Change Biology 17:2905–2935.

[CIT0017] Kraft NJ, Valencia R, Ackerly DD 2008 Functional traits and niche-based tree community assembly in an Amazonian forest. Science 322:580–582. 18948539 10.1126/science.1160662

[CIT0018] Lambers H, Poorter H 1992 Inherent variation in growth-rate between higher-plants - a search for physiological causes and ecological consequences. Advances in Ecological Research 23:187–261.

[CIT0019] Lancashire PD, Bleiholder H, Vandenboom T, Langeluddeke P, Stauss R, Weber E, Witzenberger A 1991 A uniform decimal code for growth-stages of crops and weeds. Annals of Applied Biology 119:561–601.

[CIT0020] Lefcheck JS 2016 *piecewiseSEM*: piecewise structural equation modeling in R for ecology, evolution, and systematics. Methods in Ecology and Evolution 7:573–579.

[CIT0021] Lenth RV 2016 Least-squares means: the R package *lsmeans*. Journal of Statistical Software 69:1–33.

[CIT0022] Lusk CH, Reich PB, Montgomery RA, Ackerly DD, Cavender-Bares J 2008 Why are evergreen leaves so contrary about shade?Trends in Ecology & Evolution 23:299–303. 18439708 10.1016/j.tree.2008.02.006

[CIT0023] Maire V, Wright IJ, Prentice IC, Batjes NH, Bhaskar R, van Bodegom PM, Cornwell WK, Ellsworth D, Niinemets U, Ordonez A, Reich PB, Santiago LS 2015 Global effects of soil and climate on leaf photosynthetic traits and rates. Global Ecology and Biogeography 24:706–717.

[CIT0024] Martin AR, Isaac ME 2015 Plant functional traits in agroecosystems: a blueprint for research. Journal of Applied Ecology 52:1425–1435.

[CIT0025] Martin AR, Isaac ME 2018 Functional traits in agroecology: advancing description and prediction in agroecosystems. Journal of Applied Ecology 55:5–11.

[CIT0026] Martin AR, Rapidel B, Roupsard O, Van den Meersche K, de M. Virginio Filho E, Mirna Barrios M, Isaac ME 2017 Intraspecific trait variation across multiple scales: the leaf economics spectrum in coffee. Functional Ecology 31:604–612.

[CIT0027] Meyer RS, DuVal AE, Jensen HR 2012 Patterns and processes in crop domestication: an historical review and quantitative analysis of 203 global food crops. The New Phytologist 196:29–48. 22889076 10.1111/j.1469-8137.2012.04253.x

[CIT0028] Milla R, Morente-Lopez J, Alonso-Rodrigo JM, Martin-Robles N, Chapin FS 2014 Shifts and disruptions in resource-use trait syndromes during the evolution of herbaceous crops. Proceedings of the Royal Society B 281:20141429. 10.1098/rspb.2014.1429 PMC4173681 25185998

[CIT0029] Milla R, Osborne CP, Turcotte MM, Violle C 2015 Plant domestication through an ecological lens. Trends in Ecology & Evolution 30:463–469. 26138385 10.1016/j.tree.2015.06.006

[CIT0030] Moles AT, Falster DS, Leishman MR, Westoby M 2004 Small-seeded species produce more seeds per square metre of canopy per year, but not per individual per lifetime. Journal of Ecology 92:384–396.

[CIT0031] Monfreda C, Ramankutty N, Foley JA 2008 Farming the planet: 2. Geographic distribution of crop areas, yields, physiological types, and net primary production in the year 2000. Global Biogeochemical Cycles 22: doi: 10.1029/2007GB002947

[CIT0032] Nakagawa S, Schielzeth H 2013 A general and simple method for obtaining R^2^ from generalized linear mixed-effects models. Methods in Ecology and Evolution 4:133–142.

[CIT0033] Ordoñez JC, van Bodegom PM, Witte JPM, Wright IJ, Reich PB, Aerts R 2009 A global study of relationships between leaf traits, climate and soil measures of nutrient fertility. Global Ecology and Biogeography 18:137–149.

[CIT0034] Penuelas J, Sardans J, Llusia J, Owen SM, Carnicer J, Giambelluca TW, Rezende EL, Waite M, Niinemets U 2010 Faster returns on ‘leaf economics’ and different biogeochemical niche in invasive compared with native plant species. Global Change Biology 16:2171–2185.

[CIT0035] Perez-Harguindeguy N, Diaz S, Garnier E, Lavorel S, Poorter H, Jaureguiberry P, Bret-Harte MS, Cornwell WK, Craine JM, Gurvich DE, Urcelay C, Veneklaas EJ, Reich PB, Poorter L, Wright IJ, Ray P, Enrico L, Pausas JG, de Vos AC, Buchmann N, Funes G, Quetier F, Hodgson JG, Thompson K, Morgan HD, ter Steege H, van der Heijden MGA, Sack L, Blonder B, Poschlod P, Vaieretti MV, Conti G, Staver AC, Aquino S, Cornelissen JHC 2013 New handbook for standardised measurement of plant functional traits worldwide. Australian Journal of Botany 61:167–234.

[CIT0036] Pinheiro J, Bates D, DebRoy S, Sarkar D, R Core Team. 2016 nlme: linear and nonlinear mixed effects models. R package version 3.1-127. http://CRAN.R-project.org/package=nlme.

[CIT0037] Poorter H, Niinemets U, Poorter L, Wright IJ, Villar R 2009 Causes and consequences of variation in leaf mass per area (LMA): a meta-analysis. New Phytologist 183:565–588. 10.1111/j.1469-8137.2009.02830.x 19434804

[CIT0038] Porter JR, Xie L, Challinor AJ, Cochrane K, Howden SM, Iqbal MM, Lobell DB, Travasso MI 2014 Food security and food production systems. In: Field CB, Barros VR, Dokken DJ, Mach KJ, Mastrandrea MD, Bilir TE, Chatterjee M, Ebi KL, Estrada YO, Genova RC, Girma B, Kissel ES, Levy AN, MacCracken S, Mastrandrea PR, White LL, eds. Climate change 2014: impacts, adaptation, and vulnerability. Part A: global and sectoral aspects. Contribution of working group II to the fifth assessment report of the intergovernmental panel on climate change. Cambridge, UK and New York, NY: Cambridge University Press, 485–534.

[CIT0090] R Core Team (2016). R: A language and environment for statistical computing. R Foundation for Statistical Computing, Vienna, Austria. https://www.R-project.org/.

[CIT0039] Reich PB, Ellsworth DS, Walters MB, Vose JM, Gresham C, Volin JC, Bowman WD 1999 Generality of leaf trait relationships: a test across six biomes. Ecology 80:1955–1969.

[CIT0040] Reich PB, Walters MB, Ellsworth DS 1992 Leaf life-span in relation to leaf, plant, and stand characteristics among diverse ecosystems. Ecological Monographs 62:365–392.

[CIT0041] Reich PB, Walters MB, Ellsworth DS 1997 From tropics to tundra: global convergence in plant functioning. Proceedings of the National Academy of Sciences 94:13730–13734. 10.1073/pnas.94.25.13730 PMC28374 9391094

[CIT0042] Reich PB, Wright IJ, Lusk CH 2007 Predicting leaf physiology from simple plant and climate attributes: a global GLOPNET analysis. Ecological Applications 17:1982–1988. 17974336 10.1890/06-1803.1

[CIT0043] Roucou A, Violle C, Fort F, Roumet P, Ecarnot M, Vile D 2018 Shifts in plant functional strategies over the course of wheat domestication. Journal of Applied Ecology 55:25–37.

[CIT0044] Saura-Mas S, Shipley B, Lloret F 2009 Relationship between post-fire regeneration and leaf economics spectrum in Mediterranean woody species. Functional Ecology 23:103–110.

[CIT0045] Siefert A, Violle C, Chalmandrier L, Albert CH, Taudiere A, Fajardo A, Aarssen LW, Baraloto C, Carlucci MB, Cianciaruso MV, de L Dantas V, de Bello F, Duarte LD, Fonseca CR, Freschet GT, Gaucherand S, Gross N, Hikosaka K, Jackson B, Jung V, Kamiyama C, Katabuchi M, Kembel SW, Kichenin E, Kraft NJ, Lagerström A, Bagousse-Pinguet YL, Li Y, Mason N, Messier J, Nakashizuka T, Overton JM, Peltzer DA, Pérez-Ramos IM, Pillar VD, Prentice HC, Richardson S, Sasaki T, Schamp BS, Schöb C, Shipley B, Sundqvist M, Sykes MT, Vandewalle M, Wardle DA 2015 A global meta-analysis of the relative extent of intraspecific trait variation in plant communities. Ecology Letters 18:1406–1419. 26415616 10.1111/ele.12508

[CIT0046] Van Bodegom PM, Douma JC, Witte JPM, Ordonez JC, Bartholomeus RP, Aerts R 2012 Going beyond limitations of plant functional types when predicting global ecosystem-atmosphere fluxes: exploring the merits of traits-based approaches. Global Ecology and Biogeography 21:625–636.

[CIT0047] Violle C, Navas ML, Vile D, Kazakou E, Fortunel C, Hummel I, Garnier E 2007 Let the concept of trait be functional!Oikos 116:882–892.

[CIT0048] Westoby M 1998 A leaf-height-seed (LHS) plant ecology strategy scheme. Plant and Soil 199:213–227.

[CIT0049] Westoby M, Wright IJ 2006 Land-plant ecology on the basis of functional traits. Trends in Ecology & Evolution 21:261–268. 16697912 10.1016/j.tree.2006.02.004

[CIT0050] Wood SA, Karp DS, DeClerck F, Kremen C, Naeem S, Palm CA 2015 Functional traits in agriculture: agrobiodiversity and ecosystem services. Trends in Ecology & Evolution 30:531–539. 26190137 10.1016/j.tree.2015.06.013

[CIT0051] Wright IJ, Reich PB, Cornelissen JH, Falster DS, Garnier E, Hikosaka K, Lamont BB, Lee W, Oleksyn J, Osada N, Poorter H, Villar R, Warton DI, Westoby M 2005a Assessing the generality of global leaf trait relationships. The New Phytologist 166:485–496. 15819912 10.1111/j.1469-8137.2005.01349.x

[CIT0052] Wright IJ, Reich PB, Cornelissen JHC, Falster DS, Groom PK, Hikosaka K, Lee W, Lusk CH, Niinemets U, Oleksyn J, Osada N, Poorter H, Warton DI, Westoby M 2005b Modulation of leaf economic traits and trait relationships by climate. Global Ecology and Biogeography 14:411–421.

[CIT0053] Wright IJ, Reich PB, Westoby M, Ackerly DD, Baruch Z, Bongers F, Cavender-Bares J, Chapin T, Cornelissen JH, Diemer M, Flexas J, Garnier E, Groom PK, Gulias J, Hikosaka K, Lamont BB, Lee T, Lee W, Lusk C, Midgley JJ, Navas ML, Niinemets U, Oleksyn J, Osada N, Poorter H, Poot P, Prior L, Pyankov VI, Roumet C, Thomas SC, Tjoelker MG, Veneklaas EJ, Villar R 2004 The worldwide leaf economics spectrum. Nature 428:821–827. 15103368 10.1038/nature02403

[CIT0054] Zadoks JC 1985 A decimal code for the growth-stages of cereals. Weed Research 14:415–421.

