# Supplementary material for: Inter- and intraspecific variation in leaf economic traits in wheat and maize
Source: AoB Plants. 2018 Jan 24;10(1):ply006. doi: 10.1093/aobpla/ply006 (PMC5814918; doi:10.1093/aobpla/ply006)
Supplement: Supporting Information [file ply006_suppl_supporting_information.docx]

**Supporting Information**

**Supporting Information Table S1.** Leaf functional traits employed in the studies that are incorporated into the Intergovernmental Panel on Climate Change’s 5th Assessment Report (i.e. Box 7-1, in “Projected Impacts for Crops and Livestock in Global Regions and Sub-Regions under Future Scenarios”) (Porter *et al.*, 2014). For the crop or group of crops evaluated in each individual studies Latin binomials are provided only when they were explicitly listed in the citation.

| **Citation** | **Region** | **Study Location** | **Crops** | **Latin binomial** | **Method/Model** | **Leaf Trait** |
| --- | --- | --- | --- | --- | --- | --- |
| Tao and Zhang, 2013 | East Asia | Jiangsu, Anhui and Zhejiang provinces, China | Rice | NA | Model to simulate the Crop-Weather relationship over a Large Area (MCWLA) | SLA |
| Liu *et al.* 2010 | East Asia | Huang-Huai-Hai Plain, China | Wheat-Maize | NA | Vegetation  Interface Processes (VIP) | *A*_max_ |
| Yang *et al.* 2013 | East Asia | Northern China Plain, China | Wheat | *Triticum aestivum* L. | Environmental Policy Integrated Climate (EPIC) model | - |
| Shen *et al.* 2011 | East Asia | Yangtze River, China | Rice | NA | ORYZA2000 | SLA |
| Knox *et al.* 2012 | South Asia | South Asia | Maize, Sorghum | NA | Meta-analysis | - |
| Lal, 2011 | South Asia | South Asia | Cereals | NA | Review | - |
| Srivastava *et al.* 2010 | South Asia | Central, South Central and South-West zones, India | Winter Sorghum | NA | InfoCrop-SORGHUM | SLA |
| Kumar *et al.* 2013 | South Asia | India | Rice | NA | InfoCrop-RICE | SLA |
| Byjesh *et al.* 2010 | South Asia | Mid and Upper Indo-Gangetic Plains, Southern Plateau, India | Monsoon Maize, Winter Maize | *Zea mays* L. | InfoCrop-MAIZE | SLA |
| Kumar *et al.* 2011 | South Asia | Western Ghats, Coastal Districts, Northeastern States, India | Rice, Maize, Wheat | NA | InfoCrop | SLA |
| Iqbal *et al.* 2010 | South Asia | Pakistan | Wheat, Rice | NA | Decision Support System for Agrotechnology Transfer (DSSAT) | SLA |
| Al-Bakri *et al.* 2011 | West Asia | Yarmouk Basin, Jordan | Barley, Wheat | *Triticum turgidum, Hordeum vulgare* | DSSAT | SLA |
| Knox *et al.* 2012 | Africa | Africa | Wheat, Maize, Sorghum, Millet | NA | Meta-analysis | - |
| Thornton *et al.* 2011 | Africa | Sub-Saharan Africa | Maize | NA | DSSAT | SLA |
| Thornton *et al.* 2010 | Africa | East Africa | Maize, Beans | NA | Crop Environment Resource Synthesis - CERES-Maize (DSSAT) | SLA |
| Lobell *et al.,* 2008 | Central/South America | Global (IPCC referenced areas of Northeastern Brazil, Central America, Andean Region) | Maize, Wheat, Rice | NA | Statistical Model | - |
| Costa *et al.,* 2009 | Central/South America | Southern Brazil (State of Minas Gerais) | Maize, Beans | *Zea mays* L.*, Phaseoulus vulgaris* | Model Maker | *A*_max_ |
| ECLAC, 2009 | Central/South America | Paraguay, Argentina, Central America | Wheat, Maize, Rice | NA | Review | - |
| Ruane *et al.,* 2013 | Central/South America | Panama | Maize | *Zea mays* L. | CERES-Maize (DSSAT) | SLA |
| Meza and Silva, 2009 | Central/South America | Chile | Maize, Wheat | NA | DSSAT | SLA |
| Hatfield *et al.,* 2011 | North America | Midwest, Southeast and Great Plains, US | Maize, Soy | NA | Review | - |
| Stockle *et al.,* 2010 | North America | Northwest US | Winter and Spring Wheat | NA | Cropsyst | SLA |
| Kulshreshtha, 2011 | North America | Canadian Prairies | Small grains, oil seeds | NA | Review | - |
| Iglesias *et al.,* 2012 | Europe | Boreal, Alpine, Atlantic, Continental, Mediterranean | Wheat, Maize, Soybean | NA | DSSAT | SLA |
| Luo *et al.,* 2009 | Australia | Keith, South Australia | Wheat | *Triticum aestivum* L. | Agricultural Production Systems Simulator (APSIM) | - |
| Anwar *et al.,* 2007 | Australia | Southeast Australia | Wheat | *Triticum aestivum* L. cv. Frame | Cropsyst | SLA |

**Supporting Information Table S1 References**

Al-Bakri J, Suleiman A, Abdulla F, and Ayad J. 2011. Potential impact of climate change on rainfed agriculture of a semi-arid basin in Jordan. *Physics and Chemistry of the Earth* 36: 125–134.

Anwar, MR, O’Leary G, McNeil D, Hossain H, Nelson R. 2007. Climate change impact on rainfed wheat in south-eastern Australia. Field Crops Research 104: 139–147.

Byjesh K, Kumar SN, Aggarwal PK. 2010. Simulating impacts, potential adaptation and vulnerability of maize to climate change in India. *Mitigation and Adaptation Strategies for Global Change* 15: 413–431.

Costa LC, Justino F, Oliveira LJC, Sediyama GC, Ferreira WPM, Lemos CF. 2009. Potential forcing of CO_2_, technology and climate changes in maize (*Zea mays*) and bean (*Phaseolus vulgaris*) yield in southeast Brazil. Environmental Research Letters 4: p.014013.

ECLAC. 2009. Economics of Climate Change in Latin America and the Caribbean: Summary. United Nations, Santiago, Chile.

Hatfield JL, Boote KJ, Kimball BA, Ziska LH, Izaurralde RC, Ort D, Thomson AM, Wolfe D. 2011. Climate impacts on agriculture: implications for crop production. *Agronomy Journal* 103: 351–370.

Iglesias A, Garrote L, Quiroga S, Moneo M. 2012. A regional comparison of the effects of climate change on agricultural crops in Europe. *Climatic Change* 112: 29-46.

Iqbal MM, Goheer MA, Khan AM. 2010. Climate-change aspersions on food security of Pakistan. *Science Vision* 15: 15-23.

Knox J, Hess T, Daccache A, Wheeler T. 2012. Climate change impacts on crop productivity in Africa and South Asia*. Environmental Research Letters* 7: 034032.

Kulshreshtha SN. 2011. Climate change, prairie agriculture, and prairie economy: the new normal. *Canadian Journal of Agricultural Economics* 59: 19–44.

Kumar SN, Aggarwal PK, Rani S, Jain S, Saxena R, Chauhan N. 2011. Impact of climate change on crop productivity in Western Chats, coastal and northeastern regions of India. *Current Science* 101: 332-341.

Kumar SN, Aggarwal PK, Saxena R, Rani S, Jain S, Chauhan N. 2013. An assessment of regional vulnerability of rice to climate change in India. *Climatic Change* 118: 683-699.

Lal, M. 2011. Implications of climate change in sustained agricultural productivity in South Asia. *Regional Environmental Change* 11: 79–94.

Liu S, Mo X, Lin Z, Xu Y, Ji J, Wen G, Richey J. 2010. Crop yield responses to climate change in the Huang-Huai-Hai Plain of China. *Agricultural Water Management* 97: 1195–1209.

Lobell DB, Burke MB, Tebaldi C, Mastrandrea MD, Falcon WP, Naylor RL. 2008. Prioritizing climate change adaptation needs for food security in 2030. *Science* 319: 607–610.

Luo Q, Bellotti W, Williams M, Wang E. 2009. Agriculture, ecosystems and environment adaptation to climate change of wheat growing in South Australia: analysis of management and breeding strategies. *Agriculture, Ecosystems & Environment* 129: 261–267.

Meza FJ, Silva D. 2009. Dynamic adaptation of maize and wheat production to climate change. *Climatic Change* 94: 143–156.

Ruane AC, Cecil LD, Horton RM, Gordón R, McCollum R, Brown D, Killough B, Goldberg R, Greeley AP, Rosenzweig C. 2013. Climate change impact uncertainties for maize in Panama: farm information, climate projections, and yield sensitivities. *Agricultural and Forest Meteorology* 170: 132–145.

Shen S-H, Yang S-B, Zhao Y-X, Xu Y-L, Zhao X-Y, Wang Z-Y, Liu J, Zhang W-W. 2011. Simulating the rice yield change in the middle and lower reaches of the Yangtze River under SRES B2 scenario. *Acta Ecologica Sinica* 31: 40–48.

Srivastava A, Naresh KS, Aggarwal PK. 2010. Assessment on vulnerability of sorghum to climate change in India. *Agriculture, Ecosystems & Environment* 138: 160–169.

Stöckle CO, Nelson RL, Higgins S, Brunner J, Grove G, Boydston R, Whiting M, Kruger C 2010. Assessment of climate change impact on Eastern Washington agriculture. *Climatic Change* 102: 77–102.

Tao F, Zhang Z. 2013. Climate change, high-temperature stress, rice productivity, and water use in Eastern China: a new superensemble-based probabilistic projection. *Journal of Applied Meteorology and Climatology* 52: 531–551.

Thornton PK, Jones PG, Alagarswamy G, Andresen J, Herrero, M. 2010. Adapting to climate change: agricultural system and household impacts in East Africa. *Agricultural Systems* 103: 73–82.

Thornton, PK, Jones PG, Ericksen PJ, Challinor A.J. 2011. Agriculture and food systems in sub-Saharan Africa in a 4 °C+ world. *Philosophical Transactions. Series A, Mathematical, Physical, and Engineering Sciences*, 369: 117–136.

Yang P, Wu W, Li Z, Yu Q, Inatsu M, Liu Z, Tang P, Zha Y, Kimoto M, Tang H. 2013. Simulated impact of elevated CO2, temperature, and precipitation on the winter wheat yield in the North China Plain. *Regional Environmental Change* 14: 61–74.

**Supporting Information Table S2.** Sources used to compile wheat and maize leaf trait dataset.

| **Source** | **Region** | **Country** | **Lat.** | **Long.** | **Growth Temp. (°C)** | **Total Annual Prec. (mm)** | **Spp.** | **Trait** | **Field/**  **Greenhouse/**  **Growth Chamber** | **Samp. day (days after sowing)** | **CO_2_ (ppm)** | **Nitrogen Fertilization Rate (kg N/ha)** | **Irrigation application rate (mm)** | **Obs. per study** |
| --- | --- | --- | --- | --- | --- | --- | --- | --- | --- | --- | --- | --- | --- | --- |
| Abeledo *et al.* 2014 | Agramunt | Spain | 41.79 | 1.10 | 20.5 | 333 | *Triticum aestivum* | SLA | field | 85 | - | 0, 150 | 121, 220 | 16 |
| Acciaresi & Guiamet 2010** | La Plata | Argentina | -34.00 | -58.00 | 15.4 | 0 | *Zea mays* | SLA | field | 50 | - | - | - | 6 |
| Arora *et al.* 2001 | New Delhi | India | 28.64 | 77.16 | 25.1 | 543 | *Triticum aestivum* | Leaf N | field | 85 | - | 30, 120 | - | 16 |
| Bahrun *et al.* 2003 | Copenhagen | Denmark | 56.67 | 12.30 | 11.8 | - | *Zea mays* | Leaf N | field | 78 | - | - | - | 4 |
| Balota *et al.* 2005** | Bushland | US | 35.19 | -102.06 | 25.0, 30.0 | - | *Triticum aestivum* | A*_max_* | greenhouse | 99 | - | - | - | 3 |
| Bavec *et al.* 2013 | North-eastern Slovenia | Slovenia | 43.65 | 15.68 | 19.1 | 436, 618, 809 | *Zea mays* | A*_max_* | field | 30, 85, 90 | - | 0, 70, 120, 170, 220 | - | 24 |
| Brown & Petrie 2006 | Parma | US | 43.80 | -116.94 | 15.6, 15.7 | 224, 246 | *Triticum aestivum* | Leaf N | field | 85 | - | 0, 168, 336 | - | 8 |
| Chen *et al.* 2013 | Kunming, Yunnan | China | 25.02 | 102.68 | 17.3 | 869 | *Zea mays* | A*_max_*, Leaf N | field | 82 | - | 0, 300 | - | 4 |
| Colla *et al.* 2013 | Viterbo | Italy | 42.43 | 12.09 | 19.0 | - | *Zea mays* | Leaf N | growth chamber | 28 | - | - | - | 1 |
| Condon *et al.* 1992* | New South Wales | Australia | 34.73 | 146.56 | 15.6 | 712 | *Triticum aestivum* | Leaf N, SLA | field | - | - | - | - | 4 |
| Craine *et al.* 2009* | South America | Brazil | -1.75 | -47.08 | 26.4 | 2047 | *Zea mays* | Leaf N | - | - | - | - | - | 1 |
| Creech *et al.* 2004** | Logan | US | 41.74 | -111.84 | 21.5 | - | *Zea mays* | A*_max_* | greenhouse | - | - | - | - | 1 |
| Dias *et al.* 2011 | Lisbon | Portugal | 38.70 | -9.08 | 25, 31 | - | *Triticum aestivum, Triticum durum* | A*_max_* | greenhouse | 90 | - | - | - | 8 |
| Driever *et al*. 2014** | - | UK | - | - | 15 | 1059 | *Triticum aestivum* | A*_max_* | field | 132-149 | 400 | 185 | - | 64 |
| Ecarnot *et al.* 2013 | Center, Southeast, Southwest | France | 48.82 | 2.34 | 14.7 | 605 | *Triticum durum* | Leaf N, SLA | - | - | - | 130 | - | 2 |
| Elamin & Elagib 2001 | Khartoum | Sudan | 15.23 | 32.53 | 30.9, 31.9 | - | *Zea mays* | Leaf N | field | - | - | 190 | - | 4 |
| Evans 1983 | Canberra | Australia | -35.28 | 149.13 | 16.5 | - | *Triticum aestivum* | A*_max_* | greenhouse | - | - | - | - | 5 |
| Evans, 1989* | Australia | Australia | - | - | 23.0 | - | *Triticum aestivum* | A*_max_* | growth chamber | - | - | - | - | 50 |
| Filgueira *et al.* 2003 | La Plata | Argentina | -34.00 | -58.00 | 14.0 | 958 | *Triticum aestivum* | A*_max_*, SLA | field | 86 | - | - | - | 12 |
| Gong *et al.* 2003 | Gansu Province | China | 36.05 | 103.86 | 29.5 | - | *Triticum aestivum* | SLA | greenhouse | - | - | - | - | 2 |
| Gulias *et al.* 2003* | Mallorca | Spain | 39.73 | 3.01 | 16.6 | 242 | *Zea mays* | SLA | field | - | - | - | - | 1 |
| Han *et al.* 2005* | Beijing | China | 39.80 | 116.47 | 18.8 | 410 | *Triticum aestivum, Zea mays* | Leaf N | - | - | - | - | - | 2 |
| Harrison *et al.* 2010** | Canberra | Australia | -35.20 | 149.05 | 19.3, 21.5 | 498, 429 | *Triticum aestivum* | A*_max_* | field | 168, 178 | - | 88, 160 | - | 2 |
| Hocking & Meyer 1991* | New South Wales | Australia | -34.28 | 146.06 | 21.8 | - | *Triticum aestivum, Zea mays* | Leaf N, SLA | growth chamber | 55, 58 | 360, 1500 | - | - | 20 |
| Jaikumar *et al.* 2014 | Michigan | US | 42.40 | -85.40 | 15.1 | 758 | *Triticum aestivum* | A*_max_* | field | - | - | 96 | - | 1 |
| Jin *et al.* 2015 | Beijing | China | 44.17 | 116.43 | 15.2 | 279 | *Zea mays* | A*_max_*, Leaf N | field | 51, 71, 82 | - | 0, 75, 150 | - | 54 |
| Kadam *et al.* 2015** | Los Banos | Philippines | 14.17 | 121.25 | 21.0 | - | *Triticum aestivum* | SLA | greenhouse | - | - | - | - | 4 |
| Kane *et al.* 2013 | Montreal | Canada | 45.40 | -73.56 | 5, 20 | - | *Triticum aestivum* | A*_max_* | growth chamber | NA | 380, 700 | - | - | 4 |
| Kattge *et al.* 2009* | Europe | Germany | 50.60 | 8.70 | 20.0 | 655 | *Triticum aestivum* | Leaf N, SLA | growth chamber | - | - | - | - | 20 |
| Khakwani *et al.,* 2012 | Dera Ismail Khan | Pakistan | 31.82 | 70.90 | 24.8 | - | *Triticum aestivum* | SLA | greenhouse | 90 | - | - | - | 12 |
| Lana *et al.* 2014 | Toledo | Brazil | -24.58 | -53.80 | 22.6 | 1151 | *Zea mays* | Leaf N | field | 65 | - | - | - | 2 |
| Li *et al.* 2012** | Shandong Province | China | 36.60 | 117.05 | 18.1, 19.5 | 797, 705 | *Zea mays* | A*_max_*, Leaf N | field | 82 | - | 0, 640 | - | 18 |
| Lindquist 2001 | Mead | US | 41.23 | -96.49 | 19.5 | 1145 | *Zea mays* | A*_max_* | field | 80 | - | - | - | 2 |
| Linke *et al.* 2008 | Vienna | Austria | 48.21 | 16.38 | 16.3 | - | *Triticum aestivum* | A*_max_*, Leaf N | field | 70, 85, 90 | - | 150 | - | 12 |
| Liu & Song 2012 | Jilin Province | China | 44.20 | 125.55 | 18.1 | 878 | *Zea mays* | A*_max_* | field | - | - | - | - | 7 |
| Macedo *et al.* 2003** | Lincoln | US | 40.82 | -96.69 | 21.0 | - | *Triticum aestivum* | A*_max_* | growth chamber | - | - | - | - | 1 |
| Macedo *et al.* 2005 | Bozeman | US | 45.67 | -111.05 | 21.0 | - | *Triticum aestivum* | A*_max_* | growth chamber | - | - | - | - | 2 |
| Marini *et al.* 2015** | Marechal Candido Rondon | Brazil | -24.77 | -54.37 | 22.6 | 1138 | *Zea mays* | Leaf N | field | 69 | - | 0, 40, 80, 120, 160 | - | 7 |
| Markham & Stoltenberg 2009 | Arlington | US | 43.40 | -89.30 | 15.5, 16.9 | 1002, 700 | *Zea mays* | SLA | field | 22 | - | - | - | 2 |
| Martins *et al.* 2008 | Minas Gerais | Brazil | -19.47 | -44.25 | 22.9 | 474 | *Zea mays* | Leaf N | field | 69 | - | 12, 120 | - | 2 |
| Mohammadi 2007 | Kermanshah | Iran | 34.30 | 47.08 | 18.1 | 320 | *Zea mays* | SLA | field | 128 | - | - | - | 6 |
| Moreau *et al.* 2012 | Sutton Bonington | United Kingdom | 52.83 | -1.23 | 9.1 | 690 | *Triticum aestivum* | Leaf N | field | 85 | - | - | - | 37 |
|  | Clermont Ferrand | France | 45.78 | 3.17 | 7.9, 9.7 | 588 | *Triticum aestivum* | Leaf N | field | 85 | - | - | - |  |
| Moreno-Sotomayor *et al.* 2002 | Mead | US | 41.13 | -96.50 | 16.6 | 550 | *Zea mays* | A*_max_* | field | 80, 87, 98, 120 | - | - | - | 4 |
| Oehme *et al.* 2013 | Stuttgart | Germany | 48.71 | 1.19 | 15.3, 15.4, 15.6, 16.0 | 289, 351, 383, 461 | *Triticum aestivum* | Leaf N | growth chamber | 85 | 365, 494, 529, 558, 613 | 130 | - | 8 |
| Olszewski *et al.* 2008** | Bałcyny | Poland | 53.60 | 19.85 | 12.6, 12.7, 13.2 | 307, 374, 453 | *Triticum aestivum* | A*_max_* | field | 65, 70 | - | 60, 120 | - | 36 |
|  | Montferrer | France | 41.77 | 2.58 | 14.4 | 603 | *Zea mays* | Leaf N | field | - | - | - | - |  |
| Ordonez *et al.* 2015 | Lleida | Spain | 41.73 | 0.75 | 15.9 | 399 | *Zea mays* | Leaf N | field | - | - | - | - | 3 |
|  | Algerri | Spain | 41.48 | 0.64 | 15.9 | 399 | *Zea mays* | Leaf N | field | - | - | - | - |  |
| Page *et al.* 2010** | Elora | Canada | 43.63 | -80.42 | 14.9 | 886 | *Zea mays* | SLA | field | 69 | - | - | - | 4 |
| Pal *et al.* 2005 | New Delhi | India | 28.64 | 77.16 | 25.5 | - | *Triticum aestivum* | SLA | growth chamber | 40, 60, 90 | 350, 600 | 75, 150 | - | 12 |
| Pereira *et al.* 2015 | Sao Paulo | Brazil | -20.51 | -47.39 | 25.0 | - | *Zea mays* | Leaf N | greenhouse | 60 | - | - | - | 4 |
| Pierce *et al.* 2013* | Milan | Italy | 45.78 | 8.84 | 15.7 | 548 | *Zea mays* | Leaf N, SLA | field | 128 | - | - | - | 13 |
| Ratjen & Kage 2013 | Keil | Germany | 54.19 | 9.59 | 13.9 | - | *Triticum aestivum* | SLA | field | 50, 52 | - | 0, 80, 160, 200, 240 | - | 7 |
| Rebetzke *et al.* 2004 | Canberra | Australia | -35.28 | 149.13 | 13, 19 | - | *Triticum aestivum* | SLA | field, greenhouse | 50, 60 | - | - | - | 2 |
| Reich *et al.,* 2009* | Beijing | China | 39.80 | 116.47 | 18.7 | 410 | *Zea mays* | Leaf N | - | - | - | - | - | 2 |
| Ruiz-Vera *et al.* 2015** | Champaign | US | 40.04 | -88.23 | 22.7, 22.8, 25.4, 25.5 | 868 | *Zea mays* | A*_max_* | growth chamber | - | 390, 585 | - | - | 4 |
| Sadras *et al.* 2000** | Balcarce | Argentina | -37.00 | -58.00 | 16.5, 18.7, 19.7, 23 | - | *Zea mays* | Leaf N | field | 69, 82, 96, 109 | - | 140 | - | 4 |
| Seleiman *et al. 2*013** | Helsinki | Finland | 60.23 | 25.17 | 12.8 | 725 | *Zea mays* | Leaf N | field | 75 | - | 120 | - | 6 |
| Shahbaz e*t al.* 2011 | Faisalabad | Pakistan | 31.43 | 73.09 | 31.2 | - | *Triticum aestivum* | A*_max_* | greenhouse | 91 | - | - | - | 5 |
| Shipley, 2002* | - | - | - | - | 21.0 | - | *Zea mays* | SLA | growth chamber | - | - | - | - | 1 |
| Singh *et al.* 2015 | Varanasi | India | 25.25 | 80.98 | 25.0 | 1100 | *Triticum aestivum* | Leaf N, SLA | field | - | - | 120 | - | 2 |
| Storkey 2004* | Europe | UK | 51.48 | 0.46 | 15.0 | 391, 603, 712 | *Triticum aestivum* | SLA | field | 20 | - | - | - | 4 |
| Ali Tahir *et al.* 2009 | Tottori | Japan | 35.52 | 134.26 | 23.0 | - | *Triticum aestivum* | SLA | growth chamber | - | - | - | - | 6 |
| Tausz-Posch et al. 2013** | Victoria | Australia | -36.75 | 142.11 | 9.8, 12.9 | - | *Triticum aestivum* | A*_max_* | growth chamber | 85 | 390, 550 | - | 114, 151, 174, 193 | 16 |
| Thilakarathne *et al.* 2015 | Horsham | Australia | -36.75 | 142.11 | 14.5 | 555 | *Triticum aestivum* | A*_max_*, Leaf N | growth chamber | - | 384, 550 | - | - | 8 |
| Thompson, 2015* | Mansfield | UK | 53.27 | -1.24 | 11.0 | - | *Zea mays* | SLA | - | - | - | - | - | 1 |
| Tomer *et al.* 2015 | New Delhi | India | 28.67 | 77.20 | 27.6 | 104, 312 | *Triticum aestivum, Triticum durum* | A*_max_* | growth chamber | - | - | - | - | 4 |
| Vicente *et al.* 2015 | Salamanca | Spain | 40.97 | -5.66 | 20.0 | - | *Triticum durum* | Leaf N | growth chamber | 85, 90 | 390, 700 | - | - | 4 |
| Villar *et al.* 2005 | Seville | Spain | 37.28 | -6.07 | 14.4 | 66 | *Triticum aestivum* | SLA | field | 14, 30, 43, 62 | - | - | - | 4 |
| Wang & Shangguan 2010 | Shaanxi | China | 36.51 | 109.54 | 20.9 | 375 | *Zea mays* | A*_max_* | field | 90, 150 | - | - | - | 2 |
| Wang *et al.* 2008** | Swift Current | Canada | 50.29 | -107.80 | 12.0 | 337 | *Triticum aestivum* | A*_max_*, SLA | field | 80, 90 | - | - | - | 16 |
| Xu *et al.* 2015** | Tai'an | China | 36.15 | 117.15 | 19.5, 20.0 | 569, 668 | *Triticum aestivum* | A*_max_* | field | 90, 100, 110 | - | 225 | - | 12 |
| Zhang *et al.* 2015 | Huang-Huai-Hai Plain | China | 35.02 | 114.40 | 20.1 | - | *Zea mays* | SLA | field | 82 | - | - | - | 3 |
| Zheng *et al.* 2010 | Beijing | China | 39.90 | 116.40 | 25.5 | - | *Triticum aestivum* | A*_max_* | field | 106, 113 | - | - | - | 2 |
| Zheng *et al.* 2013 | Northern China Plain | China | 36.67 | 116.37 | - | 610 | *Zea mays* | Leaf N | field | 60 | - | - | - | 2 |
| Zhou *et al.* 2015 | Flakkebjerg | Denmark | 55.32 | 11.40 | 14.1 | - | *Triticum aestivum* | A*_max_*, Leaf N | field | 47 | - | - | - | 16 |
|  | Lanoraie, Ste. Victorie, L'Acadie | Canada | 45.95 | -73.32 | 16, 17.2 | 550, 817 | *Triticum aestivum* | Leaf N | field | 54, 61, 68, 69, 74, 81, 88 | - | 0, 40, 80, 120, 160, 200, | - |  |
| Ziadi *et al.* 2010 | Ste. Victorie | Canada | 45.92 | -73.10 | 17.4 | 595 | *Triticum aestivum* | Leaf N | field | 61, 68, 76, 80 | - | 0, 40, 80, 120, 160, 200, | - | 112 |
|  | L'Acadie | Canada | 45.28 | -73.03 | 18.6, 19.1 | 439, 630 | *Triticum aestivum* | Leaf N | field | 52, 59, 58, 65, 69, 76, 85, 91, 96 | - | 0, 30, 60, 70, 90, 110, 120 | - |  |

*Data obtained via TRY

**Data obtained via graphical estimate

**Supporting Information Table S2 References**

Abeledo, L.G., Savin, R. & Slafer, G.A., 2014. Leaf photosynthesis during grain filling under Mediterranean environments: are barley or traditional wheat more efficient than modern wheats? Journal of Agronomy and Crop Science, 200(3), pp.172–182.

Acciaresi, H.A. & Guiamet, J.J., 2010. Below- and above-ground growth and biomass allocation in maize and *Sorghum halepense* in response to soil water competition. Weed Research, 50(5), pp.481–492.

Ali Tahir, I.S., Nakata, N., Yamaguchi, T., Nakano, J., & Ali, A.M.2009. Physiological response of three wheat cultivars to high shoot and root temperatures during early growth stages. Plant Production Science, 12(4), pp.409–419.

Arora, A., Singh, V.P. & Mohan, J., 2001. Effect of water stress on photosynthesis. Biologia Plantarum, 44(1), pp.153–155.

Bahrun, A., Mogensen, V.O. & Jensen, C.R., 2003. Water stress detection in field-grown maize by using spectral vegetation index. Communications in Soil Science and Plant Analysis, 34(1-2), pp.65–79.

Balota, M., Rush, C.M., Payne, W.A., & Lazar, M.D. 2005. The effect of take-all disease on gas-exchange rates and biomass in two winter wheat lines with different drought response. Plant and Soil, 275(1-2), pp.337–348.

Bavec, F., Bavec, M. & Fekonja, M., 2013. Organic and mineral nitrogen fertilizers in sweet maize (*Zea mays* L. *saccharata* Sturt.) production under temperate climate. Zemdirbyste-Agriculture, 100(3), pp.243–250.

Brown, B.D. & Petrie, S., 2006. Irrigated hard winter wheat response to fall, spring, and late season applied nitrogen. Field Crops Research, 96(2-3), pp.260–268.

Chen, J.W., Yang, Z.Q., Zhou, P., Hai, M.R., Tang, T.X. Liang, Y.L. & An, T.X. 2013. Biomass accumulation and partitioning, photosynthesis, and photosynthetic induction in field-grown maize (*Zea mays* L.) under low- and high-nitrogen conditions. Acta Physiologiae Plantarum, 35(1), pp.95–105.

Colla, G., Cardarelli, M., Rouphael, Y., Reynaud, H., Canaguier, R. & Planques, B. 2013. Effectiveness of a plant-derived protein hydrolysate to improve crop performances under different growing conditions. Acta Horticulturae, (1009), pp.175–179.

Condon, A.G., Richards, R.A. and Farquhar, G.D., 1992. The effect of variation in soil water availability, vapour pressure deficit and nitrogen nutrition on carbon isotope discrimination in wheat. Crop and Pasture Science, 43(5), pp.935-947.

Craine, J.M., Elmore, A. J., Aidar, M. P. M., Bustamante, M., Dawson, T. E., Hobbie, E. A., Kahmen, A., Mack, M.C., McLauchlan, K.K., Michelson, A., Nardato, G.B., Pardo, L.H., Penuelas, J., Reich, P.B., Schuur, E.A.G., Stock, W.D., Templer, P.H., Virginia, R.A., Wlker, J.M., & Wright, I. J.2009. Global patterns of foliar nitrogen isotopes and their relationships with climate, mycorrhizal fungi, foliar nutrient concentrations, and nitrogen availability. New Phytologist, 183(4), pp.980–992.

Creech, J.E., Monaco, T.A. & Evans, J.O., 2004. Photosynthetic and growth responses of *Zea mays* L and four weed species following post-emergence treatments with mesotrione and atrazine. Pest Management Science, 60(11), pp.1079–1084.

Driever, S.M., Lawson, T., Andralojc, P.J., Raines, C.A. and Parry, M.A.J., 2014. Natural variation in photosynthetic capacity, growth, and yield in 64 field-grown wheat genotypes. Journal of Experimental Botany, 65(17), pp.4959-4973.

Dias, A.S., Semedo, J., Ramalho, J. C., & Lidon, F. C. 2011. Bread and durum wheat under heat stress: a comparative study on the photosynthetic performance. Journal of Agronomy and Crop Science, 197(1), pp.50–56.

Ecarnot, M., Compan, F. & Roumet, P., 2013. Assessing leaf nitrogen content and leaf mass per unit area of wheat in the field throughout plant cycle with a portable spectrometer. Field Crops Research, 140, pp.44–50.

Elamin, A.E. & Elagib, M.A., 2001. Comparative study of organic and inorganic fertilizers of forage corn (*Zea mays* L.) grown on two soil types. Qatar University Science Journal, 21, pp.47–54.

Evans, J.R., 1983. Nitrogen and photosynthesis in the flag leaf of wheat (*Triticum aestivum* L.). Plant Physiology, 72(2), pp.297–302.

Evans, J.R., 1989. Photosynthesis and nitrogen relationships in leaves of C_3_ plants. Oecologia, 78(1), pp.9-19.

Filgueira, R.R., Golik, I.S., Sarli, O.G. & Jatimliansky, R.J. 2003. Anatomical and physiological characteristics of two argentine wheat cultivars. Ciência Rural, 33(4), pp.641–645.

Gong, H., Chen, K.M., Chen, G.C., Wang, S.M. & Zhang, C.L 2003. Effects of silicon on growth of wheat under drought. Journal of Plant Nutrition, 26(5), pp.37–41.

Gulias, J., Flexas, J., Mus, M., Cifre, J., Lefi, E., & Medrano, H. 2003. Relationship between maximum leaf photosynthesis, nitrogen content and specific leaf area in Balearic endemic and non-endemic Mediterranean species. Annals of Botany, 92(2), pp.215–222.

Han, W., Fang, J., Guo, D. & Zhang, Y. 2005. Leaf nitrogen and phosphorus stoichiometry across 753 terrestrial plant species in China. New Phytologist, 168(2), pp.377–385.

Harrison, M.T., Kelman, W.M., Moore, A.D. & Evans, J.R. 2010. Grazing winter wheat relieves plant water stress and transiently enhances photosynthesis. Functional Plant Biology, 37(8), pp.726–736.

Hocking, P. & Meyer, C., 1991. Enrichment and nitrogen stress on growth, and partitioning of dry matter and nitrogen in wheat and maize. Australian Journal of Plant Physiology, 18(4), p.339.

Jaikumar, N.S., Snapp, S.S., Flore, J.A. & Loescher, W. 2014. Photosynthetic responses in annual rye, perennial wheat, and perennial rye subjected to modest source: sink ratio changes. Crop Science, 54(1), pp.274–283.

Jin, X., Yang, G, Tan, C. & Zhao, C. 2015. Effects of nitrogen stress on the photosynthetic CO2 assimilation, chlorophyll fluorescence, and sugar-nitrogen ratio in corn. Scientific Reports, 5, p.9311.

Kadam, N.N., Yin, X., Bindraban, P., Struik, P.C. & Jagadish, K.S.V. 2015. Does morphological and anatomical plasticity during the vegetative stage make wheat more tolerant of water deficit stress than rice? Plant Physiology, 167(4), pp.1389–1401.

Kane, K., Dehal, K.P., Badawi, M.A., Houde, M., Huner, N.P.A. & Sarhan, F. 2013. Long-term growth under elevated CO_2_ suppresses biotic stress genes in non-acclimated, but not cold-acclimated winter wheat. Plant and Cell Physiology, 54(11), pp.1751–1768.

Kattge, J., Diaz, S., Lavorel, S., Prentice, C., Leadley, P., Bonisch, G., Garnier, E., Westoby, M., Reich, P.B., Wright, I.J., Cornelissen, J.H.C., Violle, C., Harrison, S.P., van Bodegom, P.M., Reichstein, M., Enquist, B.J., Soudzilovskaia, N.A., Ackerly, D.D., Anand, M., Atkin, O., Bahn, M., Baker, T.R., Baldocchi, D., Bekker, R., Blanco, C.C., Blonder, B., Bond, W.J., Bradstock, R., Bunker, D.E., Casanoves, F., Cavender-Bares, J., Chamber, J.Q., Chapin, F.S., Chave, J., Coomes, D., Cornwell, W.K., Craine, J.M., Dobrin, B.H., Duarte, L., Durka, W., Elser, J., Esser, G., Estiarte, M., Fagan, W.F., Fang, J., Fernandez-Mendez, F., Fidelis, Finegan, B., Flores, O., Ford, H., Frank, D., Freschet, G.T., Fyllas, N.M., Gallagher, R.V., Green, W.A., Gutierrez, A.G., Hickler, T., Higgins, S.I., Hodgson, J.G., Jalili, A., Jansen, S., Joly, C.A., Kerkhoff, A.J., Kirkup, D., Kitajima, K., Kleyer, M., Klotz, S., Knops, J.M.H., Kramer, K., Kuhn, I., Kurokawa, H., Laughlin, D., Lee, T.D., Leishman, M., Lens, F., Lenz, T., Lewis, S.L., Llyod, J., Llusio, J., Louault, F., Ma, S., Mahecha, A.T., Muller, S.C., Nadrowski, K., Naeem, S., Niinemets, U., Nollert, S., Nuske, A., Ogaya, R., Oleksyn, J., Onipchenko, V.G., Onoda, Y., Ordonez, J., Overbeck, G., Ozinga, W.A., Patino, S., Paula, S., Pausas, J.G., Penuelas, J., Phillips, O.L, Pillar, V., Poorter, H., Poorter, L., Poschlod, P., Prinzing, A., Proulx, R., Rammig, A., Reinsch, S., Reu, B., Sack, L., Salgado-Negret, B., Sardans, J., Shiodera, S., Shipley, B., Siefert, A., Sosinski, E., Soussana, J.F., Swaine, E., Swenson, N., Thompson, K., Thornton, P., Waldram, M, Weiher, E., White, M., White, S., Wright, S.J., Yguel, B., Zaehle, S., Zanne, A.E., and Wirth, C. 2009. Quantifying photosynthetic capacity and its relationship to leaf nitrogen content for global-scale terrestrial biosphere models. Global Change Biology, 15(4), pp.976–991.

Khakwani, A. A., Dennett, M. D., Munir, M., & Baloch, M. S. (2012). Wheat yield response to physiological limitations under water stress condition. The Journal of Animal & Plant Sciences, 22, 773-780.

Lana, M. do C., Rampim, L., Ohland, T., & Fávero, F. 2014. Spacing, population density and nitrogen fertilization in corn grown in an Oxisoil. Revista Ceres, 61(3), pp.424–433.

Li, G., Zhang, Z. S., Gao, H. Y., Liu, P., Dong, S. T., Zhang, J. W., and Zhao, B.2012. Effects of nitrogen on photosynthetic characteristics of leaves from two different stay-green corn (*Zea mays* L.) varieties at the grain-filling stage. Canadian Journal of Plant Science, 92(4), pp.671–680.

Lindquist, J.L., 2001. Light-saturated CO_2_ assimilation rates of corn and velvetleaf in response to leaf nitrogen and development stage. Weed Science, 49(6), pp.706–710.

Linke, R., Richter. K., Haumann, J., Schneider, W. & Weihs, P. 2008. Occurrence of repeated drought events: can repetitive stress situations and recovery from drought be traced with lead reflectance? Periodicum Biologorum, 110(3), pp.219–229.

Liu, T.D. & Song, F.B., 2012. Maize photosynthesis and microclimate within the canopies at grain-filling stage in response to narrow-wide row planting patterns. Photosynthetica, 50(2), pp.215–222.

Macedo, T.B., Higley, L.G., Ni, X. & Quisenberry, S.S. 2003. Light activation of Russian wheat aphid-elicited physiological responses in susceptible wheat. Journal of economic entomology, 96(1), pp.194–201.

Macedo, T.B, Peterson, R.K.D., Weaver, D.K. & Morrill, W.L. 2005. Wheat stem sawfly, *Cephus cinctus* Norton, impact on wheat primary metabolism: an ecophysiological approach. Environmental Entomology, 34(3), pp.719–726.

Marini, D., Vandeir, F.G., Dartora, J., de Carmos Lana, & M., Pinto Jr., A.S. 2015. Growth and yield of corn hybrids in response to association with *Azospirillum brasilense* and nitrogen fertilization. Revista Ceres, pp.117–123.

Markham, M.Y. & Stoltenberg, D.E., 2009. Red:far-red light effects on corn growth and productivity in field environments. Weed Science, 57(2), pp.208–215.

Martins, A.O.. Campostrini, E., Magalhães, P.C., Guimarães, L.J.M., Durães, F.O.M., Marriel, I.E. & Netto A.T. 2008. Nitrogen-use efficiency of maize genotypes in contrasting environments. Crop Breeding and Applied Biotechnology, 8, pp.291–298.

Mohammadi, G.R., 2007. Growth parameters enhancing the competitive ability of corn (*Zea mays* L.) against weeds. Weed Biology and Management, 7(4), pp.232–236.

Moreau, D., Allard, V., Gaju, O., Le Gouis, J., Foulkes, M. J., and Martre, P. 2012. Acclimation of leaf nitrogen to vertical light gradient at anthesis in wheat is a whole-plant process that scales with the size of the canopy. Plant Physiology, 160(3), pp.1479–90.

Moreno-Sotomayor, A., Weiss, A., Paparozzi, E.T., & Arkebauer, T.J., 2002. Stability of leaf anatomy and light response curves of field grown maize as a function of age and nitrogen status. Journal of Plant Physiology, 159, pp.819–826.

Oehme, V., Högy, P., Franzaring, J., Zebitz, C.P.W., and Fangmeier, A., 2013. Pest and disease abundance and dynamics in wheat and oilseed rape as affected by elevated atmospheric CO_2_ concentrations. Functional Plant Biology, 40(2), pp.125–136.

Olszewski, J., Pszczółkowska, A., Kulik, T., Fordoński, G., Płodzień, K., Okorski, A. & Wasielewska, J.2008. Rate of photosynthesis and transpiration of winter wheat leaves and ears under water deficit conditions. Polish Journal of Natural Science, 23(2), pp.326–335.

Ordonez, R.A., Savin, R. & Slafer, G.A., 2015. Genetic variation in the critical specific leaf nitrogen maximising yield among modern maize hybrids. Field Crops Research, 172, pp.99–105.

Page, E.R., Tollenaar, M., Lee, E.A., Lukens, L., & Swanton, C.J., 2010. Timing, effect, and recovery from intraspecific competition in maize. Agronomy Journal, 102(3), pp.1007–1013.

Pal, M., Rao, L.S., Jain, V., Srivastava, A.C., Pandey, R., Raj, A., & Singh, K.P., 2005. Effects of elevated CO_2_ and nitrogen on wheat growth and photosynthesis. Biologia Plantarum, 49(3), pp.467–470.

Pereira, L. de M., Revolti, L.T.M., Zingaretti, S.M., & Môro, G.V., 2015. Seed quality, chlorophyll content index and leaf nitrogen levels in maize inoculated with *Azospirillum brasilense*. Revista Ciencia Agronomica, 46(3), pp.630–637.

Pierce, S., Brusa, G., Vagge, I., & Cerabollini, B.E.L., 2013. Allocating CSR plant functional types: the use of leaf economics and size traits to classify woody and herbaceous vascular plants. Functional Ecology, 27(4), pp.1002–1010.

Ratjen, A.M. & Kage, H., 2013. Is mutual shading a decisive factor for differences in overall canopy specific leaf area of winter wheat crops? Field Crops Research, 149, pp.338–346.

Rebetzke, G.J., Botwright, T.L., Moore, C.S., Richards, R.A., & Condon, A.G., 2004. Genotypic variation in specific leaf area for genetic improvement of early vigour in wheat. Field Crops Research, 88(2-3), pp.179–189.

Reich, P. B., J. Oleksyn, and I. J. Wright. 2009. Leaf phosphorus influences the photosynthesis-nitrogen relation: a cross-biome analysis of 314 species. Oecologia 160:207-212

Ruiz-Vera, U.M., Sibers, M.H., Drag, D.W., Ort, D.R., & Bernacchi, C.J., 2015. Canopy warming caused photosynthetic acclimation and reduced seed yield in maize grown at ambient and elevated CO_2_. Global Change Biology, 21(11), pp.4237–4249.

Sadras, V., Echarte, L. & Andrade, F., 2000. Profiles of leaf senescence during reproductive growth of sunflower and maize. Annals of Botany, 85, pp.187–195.

Seleiman, M.F., Santanen, A., Jaakkola, S., Ekholm, P., Hartikainen, H., Stoddard, F.L., & Mäkelä, P.S.A., 2013. Biomass yield and quality of bioenergy crops grown with synthetic and organic fertilizers. Biomass and Bioenergy, 59, pp.477–485.

Shahbaz, M., Masood, Y., Perveen, S., & Ashraf, M., 2011. Is foliar-applied glycinebetaine effective in mitigating the adverse effects of drought stress on wheat (*Triticum aestivum* L.)? Journal of Applied Botany and Food Quality, 84(2), pp.192–199.

Shipley B., 2002. Trade-offs between net assimilation rate and specific leaf area in determining relative growth rate: relationship with daily irradiance. Functional Ecology(16) 682-689

Singh, V., Gupta, S., Singh, H., & Raghubanshi, A.S., , 2015. Ecophysiological characteristics of five weeds and a wheat crop in the Indo-Gangetic Plains, India. Weed Biology and Management, 15(3), pp.102–112.

Storkey, J., 2004. Modelling seedling growth rates of 18 temperate arable weed species as a function of the environment and plant traits. Annals of Botany, 93(6), pp.681–689.

Tausz-Posch, S., Borowiak, K., Dempsey, R.W., Norton, R.M., Seneweera, S., Fitzgerald, G.J., & Tausz, 2013. The effect of elevated CO_2_ on photochemistry and antioxidative defence capacity in wheat depends on environmental growing conditions - A FACE study. Environmental and Experimental Botany, 88, pp.81–92.

Thilakarathne, C.L., Tausz-Posch, S., Cane, K., Norton, R.M., Fitzgerald, G.J., Tausz, M., & Seneweera, S., 2015. Intraspecific variation in leaf growth of wheat (*Triticum aestivum*) under Australian Grain Free Air CO_2_ Enrichment (AGFACE): is it regulated through carbon and/or nitrogen supply? Functional Plant Biology, 42(3), pp.299–308.

Thompson, K. 2015. Personal communication. Received data via TRY database, contacted for supplementary information.

Tomer, R., Bhatia, A., Kumar, V., Kumar, A., Singh, R., Singh, B., & Singh, S.D., 2015. Impact of elevated ozone on growth, yield and nutritional quality of two wheat species in northern India. Aerosol and Air Quality Research, 3, pp.329–340.

Vicente, R., Pérez, P., Martínez-Carrasco, R., Gutiérrez, E., & Morcuende, R., 2015. Nitrate supply and plant development influence nitrogen uptake and allocation under elevated CO_2_ in durum wheat grown hydroponically. Acta Physiologiae Plantarum, 37(6), p.114.

Villar, R., Marañón, T., Quero, J.L., Panadero, P., Arenas, F., & Lambers, H., 2005. Variation in relative growth rate of 20 *Aegilops* species (Poaceae) in the field: the importance of net assimilation rate or specific leaf area depends on the time scale. Plant and Soil, 272(1-2), pp.11–27.

Wang, H., McCaig, T.N., DePauw, R.M., & Clarke, J.M., 2008. Flag leaf physiological traits in two high-yielding Canada Western Red Spring wheat cultivars. Canadian Journal of Plant Science, 88(1), pp.35–42.

Wang, K. & Shangguan, Z., 2010. Photosynthetic characteristics and resource utilization efficiency of maize (*Zea mays* L.) and millet (*Setaria italica* L.) in a semi-arid hilly loess region in China. New Zealand Journal of Crop and Horticultural Science, 38(4), pp.247–254.

Xu, H.C., Cai, T., Wang, Z.L., & Wing-Rong, H.E., 2015. Physiological basis for the differences of productive capacity among tillers in winter wheat. Journal of Integrative Agriculture, 14(10), pp.1958–1970.

Zhang, C., Zhang, J., Zhang, H.Z., Zhao, J., Wu, Q., Zhao, Z. & Cai, T.2015. Mechanisms for the relationships between water-use efficiency and carbon isotope composition and specific leaf area of maize (Zea mays L.) under water stress. Plant Growth Regulation, 77(2), pp.233–243.

Zheng, Y., Xu, X., Simmons, M., Zhang, C., Gao, F., & Li, Z., 2010. Responses of physiological parameters, grain yield, and grain quality to foliar application of potassium nitrate in two contrasting winter wheat cultivars under salinity stress. Journal of Plant Nutrition and Soil Science, 173(3), pp.444–452.

Zheng, Y., Xu., M., Shen, R., & qui, S., 2013. Effects of artificial warming on the structural, physiological, and biochemical changes of maize (*Zea mays* L.) leaves in northern China. Acta Physiologiae Plantarum, 35(10), pp.2891–2904.

Zhou, Q., Ravnskov, S., Jiang, D., & Wollenweber, B., 2015. Changes in carbon and nitrogen allocation, growth and grain yield induced by arbuscular mycorrhizal fungi in wheat (*Triticum aestivum* L.) subjected to a period of water deficit. Plant Growth Regulation, 75(3), pp.751–760.

Ziadi, N., Bélanger, G., Claessens, A., Lefebvre, L., Tremblay, N., Cambouris, A.N., Nolin, M.C., and Parent, L.-É., 2010. Plant-based diagnostic tools for evaluating wheat nitrogen status. Crop Science, 50(6), pp.2580–2590.

**Supporting Information Table S3.** Evaluation of intraspecific trait variation in three leaf traits in wheat and maize data, as a function of nitrogen fertilization (N), irrigation (I), type of study (F), and number of days since planting (D). Parameters were fit assuming a random effect associated with the source publication (P). Parameters here, and the detailed model structure is presented in Equation 1 in the main text. Significant model terms (where *p*≤0.05) are highlighted in bold.

| **Trait** | **Model parameter** | **Num.**  **D.F.** | **Den.**  **D.F.** | ***F*-value** | ***p*-value** |
| --- | --- | --- | --- | --- | --- |
| Specific leaf area | **Intercept** | **1** | **48** | **447.77** | **<0.0001** |
|  | N | 1 | 48 | 0.64 | 0.427 |
|  | **I** | **1** | **48** | **14.19** | **0.001** |
|  | **F** | **3** | **48** | **5.44** | **0.003** |
|  | **D** | **1** | **48** | **44.36** | **<0.0001** |
| Leaf N | **Intercept** | **1** | **45** | **196.47** | **<0.0001** |
|  | **N** | **1** | **45** | **16.46** | **0.0002** |
|  | I | 1 | 45 | 0.02 | 0.901 |
|  | F | 2 | 15 | 1.16 | 0.341 |
|  | D | 1 | 45 | 0.31 | 0.584 |
| *A*_max_ | **Intercept** | **1** | **12** | **30.96** | **<0.0001** |
|  | N | 1 | 12 | 2.63 | 0.131 |
|  | I | 1 | 12 | 0.17 | 0.688 |
|  | F | 1 | 6 | 0.65 | 0.45 |
|  | **D** | **1** | **12** | **5.11** | **0.043** |

**Supporting Information Table S4.** Linear mixed effects models predicting leaf traits as a function of growth temperature (GT), total annual precipitation (TAP), species identity (S), and interactions terms (denoted by “*”). Also shown are corresponding model AIC values, with ΔAIC values corresponding to differences between a reduced model relative to the full model (i.e. model 5), as well as the variance in each trait explained by the fixed effects terms in the model (“Marginal *r*^2^”), and the variance explained by both the fixed effects and random effects (“Conditional *r*^2^”). Random effects were based on preliminary analysis (see Equation 1 in the main text and Supporting Information Table S3), and included the number of days since planting for *A*_max_, nitrogen fertilization only for leaf N, and the number of days since planting, irrigation, and type of study for SLA.

| Trait | Model | Fixed effects terms | AIC | ΔAIC | Marginal *r*^2^ | Conditional *r*^2^ |
| --- | --- | --- | --- | --- | --- | --- |
| *A*_max_ (*n*=197) | 1 | GT + TAP | 1267.5 | 40.0 | 0.37 | 0.78 |
|  | 2 | GT + TAP + S | 1245.8 | 17.2 | 0.472 | 0.736 |
|  | 3 | GT + TAP + S + S*GT | 1231.4 | 2.8 | 0.465 | 0.734 |
|  | 4 | GT + TAP + S + S*TAP | 1233.5 | 4.9 | 0.389 | 0.73 |
|  | 5 | GT + TAP + S + S*GT + S*TAP | 1228.6 | 0 | 0.394 | 0.73 |
| Leaf N (*n*=206) | 1 | GT + TAP | 1497.3 | -0.03 | 0.051 | 0.361 |
|  | 2 | GT + TAP + S | 1498.8 | 1.4 | 0.056 | 0.354 |
|  | 3 | GT + TAP + S + S*GT | 1499.1 | 1.7 | 0.054 | 0.364 |
|  | 4 | GT + TAP + S + S*TAP | 1499.7 | 2.4 | 0.061 | 0.356 |
|  | **5** | GT + TAP + S + S*GT + S*TAP | 1497.4 | 0 | 0.069 | 0.38 |
| SLA (*n*=34) | 1 | GT + TAP | 187.2 | 0.7 | 0.437 | 0.966 |
|  | 2 | GT + TAP + S | 184.7 | -1.9 | 0.458 | 0.959 |
|  | 3 | GT + TAP + S + S*GT | 185.8 | -0.7 | 0.631 | 0.974 |
|  | 4 | GT + TAP + S + S*TAP | 184.6 | -2.0 | 0.455 | 0.948 |
|  | 5 | GT + TAP + S + S*GT + S*TAP | 186.5 | 0 | 0.528 | 0.959 |

**Supporting Information Table S5.** Variation in three functional traits in relation to growth temperature and precipitation. In these models all fixed effects were included (based on the results of an AIC model comparison (see Table S4)), which included species identity (S), growth temperature (GT), total annual precipitation (TAP), and associated interaction terms (denotes by “*”). In these models, *Z*. *mays* was coded as a dummy variable in order to evaluate differences among species (S). Therefore, any parameters that include “S” are associated with *Z*. *mays* only. Significant model parameters (where *p* ≤ 0.05) are highlighted in bold. Random effects were also included in these models based on preliminary analysis (see Equation 1 and Supporting Information Table S3). Specifically, across the three different models random effects included i) the number of days since planting for *A*_max_, ii) nitrogen fertilization only for leaf N, iii) and the number of days since planting, irrigation, and type of study for SLA.

| Trait | Parameter | Estimate | s.e. | d.f. | *t*-value | *p*-value |
| --- | --- | --- | --- | --- | --- | --- |
| *A*_max_ (*n*=197) | **Intercept** | **-13.41** | **6.49** | **161** | **-2.07** | **0.04** |
|  | **S** | **48.89** | **11.26** | **161** | **4.34** | **<0.001** |
|  | **GT** | **0.93** | **0.38** | **161** | **2.43** | **0.016** |
|  | **TAP** | **0.02** | **0.003** | **161** | **7.93** | **<0.001** |
|  | **S*GT** | **-1.82** | **0.7** | **161** | **-2.61** | **0.01** |
|  | **S*TAP** | **-0.01** | **0.01** | **161** | **-2.29** | **0.023** |
| Leaf N (*n*=206) | **Intercept** | **43.6** | **6.8** | **199** | **6.37** | **<0.001** |
|  | S | -14.3 | 9.0 | 199 | -1.59 | 0.114 |
|  | **GT** | **-1.2** | **0.3** | **199** | **-4.4** | **<0.001** |
|  | **TAP** | **0.01** | **0.005** | **199** | **2.47** | **0.015** |
|  | **S*GT** | **1.2** | **0.6** | **199** | **2.09** | **0.038** |
|  | S*TAP | -0.01 | 0.007 | 199 | -1.91 | 0.057 |
| SLA (*n*=34) | **Intercept** | **48.26** | **8.87** | **19** | **5.44** | **<0.001** |
|  | S | 20.73 | 35.07 | 6 | 0.59 | 0.576 |
|  | **GT** | **-1.51** | **0.44** | **19** | **-3.42** | **0.003** |
|  | **TAP** | **-0.02** | **0.004** | **19** | **-4.43** | **0.0003** |
|  | S*GT | -0.97 | 2.15 | 19 | -0.45 | 0.659 |
|  | S*TAP | 0.01 | 0.01 | 19 | 0.74 | 0.471 |


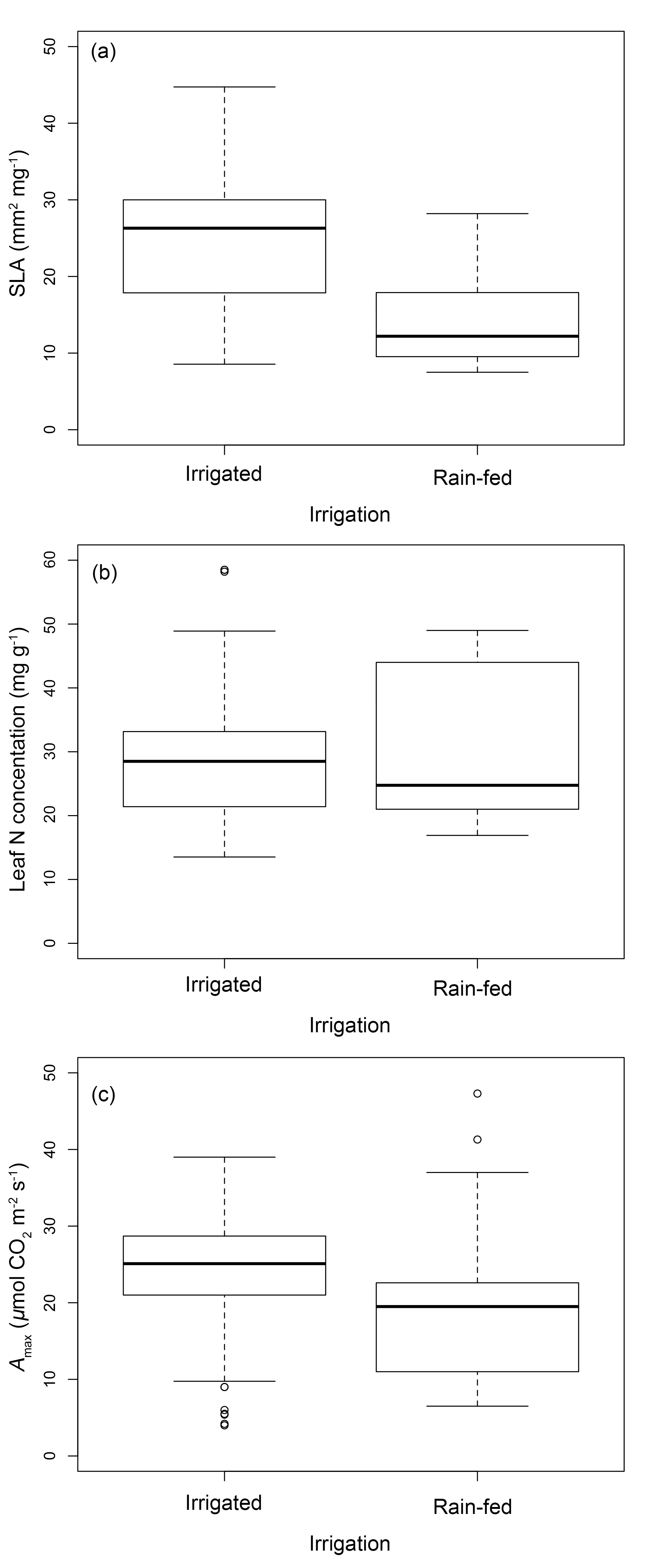


**Supporting Information Figure S1.** Differences in leaf three leaf traits in wheat and maize across two irrigation treatments. Sample sizes in irrigated and rain-fed systems were as follows: a) SLA, *n*=77 and *n*=20, respectively; b) leaf N concentrations, *n*=88 and *n*=10, respectively; and c) *A*_max_, *n*=101 and *n*=28, respectively. Specific leaf area differed significantly across treatments (*t*_31.15_=5.43, *p*<0.001), while differences in leaf N (*t*_10.46_=-0.41, *p*=0.691), and *A*_max_ among treatments were not significantly different (*t*_35.09_=1.82, *p*=0.077).


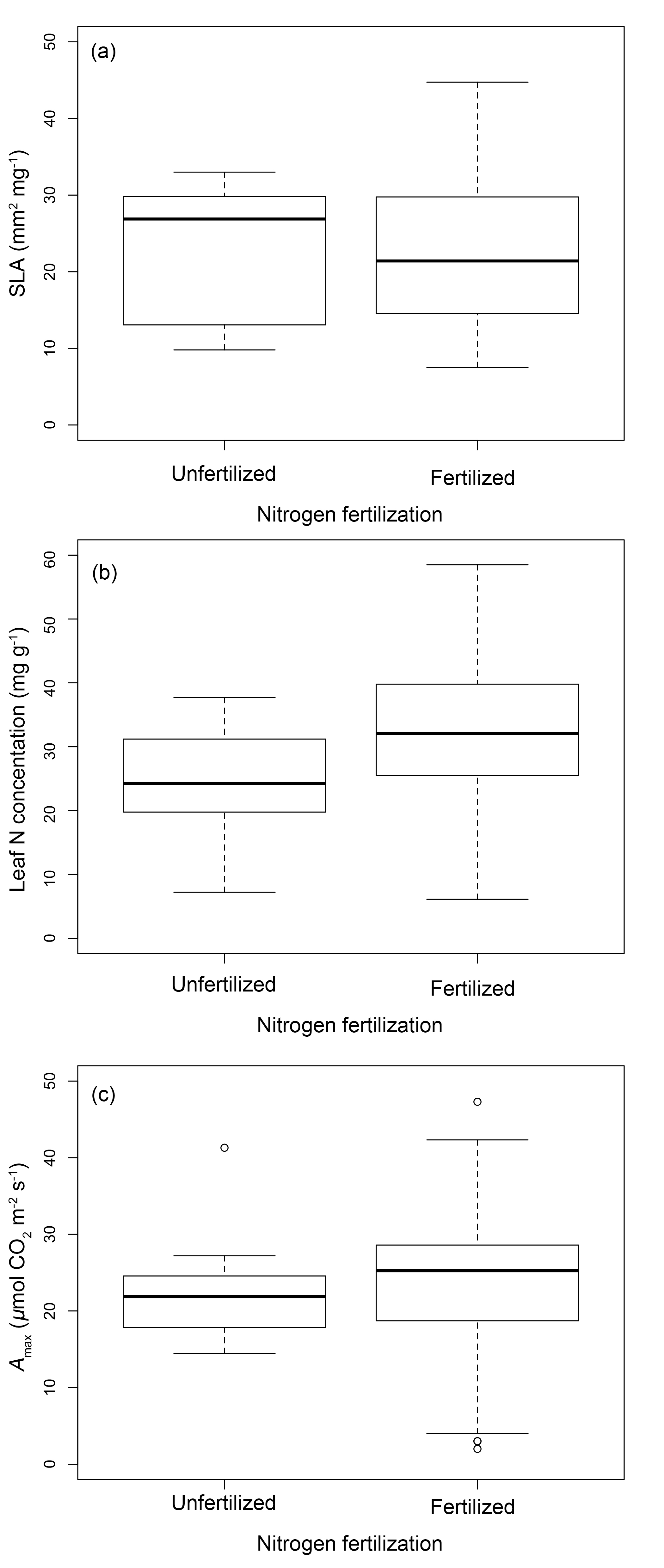


**Supporting Information Figure S2.** Differences in leaf three leaf traits in wheat and maize across two irrigation treatments. Sample sizes in unfertilized and fertilized treatments were as follows: a) SLA, *n*=19 and *n*=78, respectively; b) leaf N concentrations, *n*=40 and *n*=210, respectively; and c) *A*_max_, *n*=20 and *n*=240, respectively. Both SLA and *A*_max_ did not differ significantly across treatments (SLA *t*_26.55_=0.1, *p*=0.923, *A*_max_ *t*_24.47_=-1.07, *p*=0.296), while differences in leaf N across treatments were statistically significant (*t*_66.0_=-5.77, *p*<0.001).


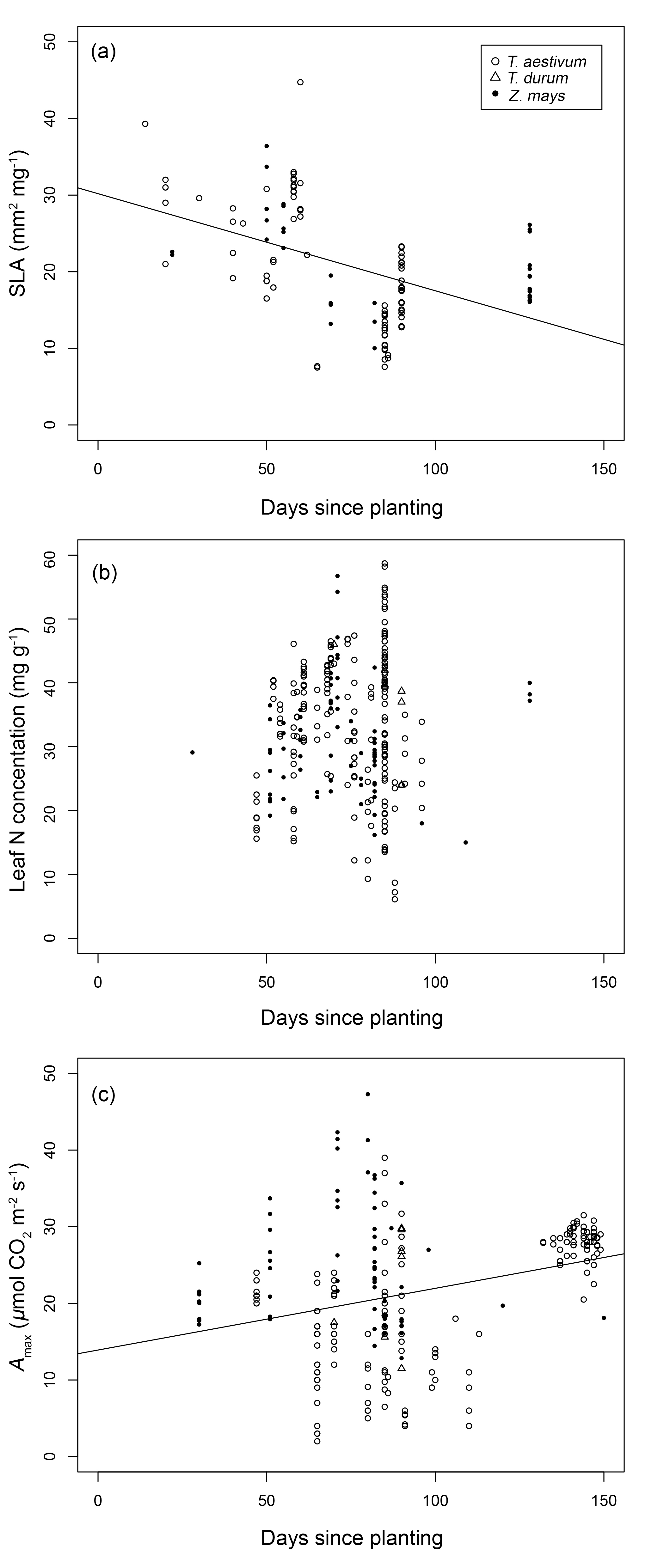


**Supporting Information Figure S3.** Variation in leaf three leaf traits as a function of the numbers of days since planting. Symbols correspond to species identity, and total sample sizes are as follows: a) SLA, *n*=244; b) leaf N concentrations, *n*=284; and c) *A*_max_, *n*=116. Trend lines represent statistically significant simple linear regression models for SLA (*r*^2^=0.234, *p*<0.001) and *A*_max_ (*r*^2^=0.101, *p*=0.001). The Non-significant linear relationship for leaf N is not shown (*r*^2^=0.002, *p*=0.423).
